# Interaction of SERINC5 and IFITM1/2/3 regulates the autophagy-apoptosis-immune network under CSFV infection

**DOI:** 10.1080/21505594.2022.2127241

**Published:** 2022-10-07

**Authors:** Wenhui Li, Zilin Zhang, Liangliang Zhang, Qingfeng Zhou, Yuwan Li, Lin Yi, Hongxing Ding, Mingqiu Zhao, Jinding Chen, Shuangqi Fan

**Affiliations:** a College of Veterinary Medicine, South China Agricultural University, Guangzhou, China; b Key Laboratory of Zoonosis Prevention and Control of Guangdong Province, South China Agricultural University, Guangzhou, China; c Key Laboratory of Animal Vaccine Development, Ministry of Agriculture and Rural Affairs, Guangzhou, China; d College of Food Science and Engineering, Shandong Agricultural University, Taian, China; e Wen’ s Foodstuffs Group Co, Ltd, Guangdong, China

**Keywords:** CSFV, SERINC5, IFITM1/2/3, autophagy, apoptosis

## Abstract

The host restriction factor serine incorporator 5 (SERINC5) plays a key role in inhibiting viral activity and has been shown to inhibit classical swine fever virus (CSFV) infection. However, the action of SERINC5 in the interaction between host cells and CSFV remains poorly understood. This study found that SERINC5 represses CSFV-induced autophagy through MAPK1/3-mTOR and AKT-mTOR signalling pathways. Further research showed that SERINC5 promotes apoptosis by repressing autophagy. Likewise, it was demonstrated that SERINC5 interacting proteins IFITM1/2/3 inhibit CSFV replication and regulate autophagy in a lysosomal-associated membrane protein LAMP1-dependent manner. In addition, IFITM1/2/3 interference promotes the NF-κB signalling pathway for potential immunoregulation by inhibiting autophagy. Finally, the functional silencing of IFITM1/2/3 genes was demonstrated to enhance the inhibitory effect of SERINC5 on autophagy. Taken together, These data uncover a novel mechanism through SERINC5 and its interacting proteins IFITM1/2/3, which mediates CSFV replication, and provides new avenues for controlling CSFV.

## Introduction

Classical swine fever is a highly contagious and economically significant viral disease, which causes haemorrhagic necrotizing inflammation, high fever, as well as respiratory and gastrointestinal symptoms [1–3]. Classical swine fever virus (CSFV) is the causative agent of classical swine fever; its genome has a length of about 12.3 kb and encodes eight non-structural and four structural proteins [3–6]. The main characteristics of CSFV are immunosuppression and multicellular infection, infecting pig testis cells, porcine alveolar macrophages, pig kidney cells, as well as dendritic cells [7–10]. Although primary studies of CSFV pathogenesis have been conducted in pigs, the precise molecular mechanism of CSFV pathogenesis remains largely unknown [11].

Viral pathogenicity is closely related to cellular responses, which regulating cell homoeostasis (e.g. autophagy, apoptosis, pyroptosis, and necrosis) to promote or inhibit virus replication [12]. Among these responses, autophagy is a recycling process of eukaryotic cells via evolutionarily conserved cell degradation. The substrates to be degraded (e.g. damaged proteins and organelles) are wrapped by double-membrane vesicles, thus forming autophagosomes. These then fuse with lysosomes, forming autolysosomes for the degradation process and ultimately, the recycling of the endocytosed cellular components of the substrate [13–15]. Three main types of molecular mechanisms are employed by substrate targeting lysosomes: microautophagy, macroautophagy, and chaperon-mediated autophagy [16,17]. Our previous studies showed that CSFV regulates autophagy via virion release from host cells and enhances viral replication [18,19]. It has also been shown that CSFV infection promotes proliferation through autophagy-related pathways such as mitophagy [20], stress-induced autophagy in the endoplasmic reticulum [21], autophagy inhibiting apoptosis [19], and autophagy inhibiting type I IFN secretion [22]. In addition, it has been suggested that autophagy affects CSFV replication through interaction with host proteins [23,24]. However, how the host affects CSFV replication through the autophagy pathway remains unclear.

During the process of viral infection, host cells express numerous anti-viral proteins, which utilize the innate immune system to resist persistent infection and proliferation of the virus [25]. In recent years, many host innate immune response mechanisms and host cell proteins related to anti-virus action by the host have been reported [26–28]. The host cell protein serine incorporator 5 (SERINC5) is a transmembrane protein with antiviral effect that inhibits both viral infection and virus fusion early in the viral replication cycle [29,30]. SERINC5 has high anti-retroviral activity against viruses, such as murine leukaemia virus, human immunodeficiency virus, and simian immunodeficiency virus [31–33]. Recent studies have shown that SERINC5 has broad-spectrum antiviral activity and may inhibit enveloped viruses except for retroviruses [34,35]. Our previous study showed that SERINC5 has anti-CSFV activity [36], but the specific mechanism of SERINC5 in the interaction between CSFV and host cells remains unclear.

Interferon-induced transmembrane proteins (IFITMs) are a family of small homologous proteins, which involved in antiviral responses to a variety of viruses, including African swine fever virus [37–39], influenza A virus [40], SARS coronavirus [38], and vesicular stomatitis virus [41]. Recent studies showed that IFITM1/2/3 proteins inhibit the proliferation of CSFV, which may be related to lysosomes [42]. Moreover, IFITM3 is involved in the antiviral response mechanism of cells, where it mediates the RNA-virus-triggered induction of type I IFN by regulating autophagy degradation of IRF3 [43]. Despite the involvement of IFITMs in CSFV replication, the specific role of IFITMs in CSFV replication remains poorly understood.

In this study, the mechanism of the host cell protein SERINC5 and its interacting proteins IFITM1/2/3 use to regulate the autophagy-apoptosis-immune network under CSFV infection was investigated. The results showed that SERINC5 inhibits CSFV-induced autophagy through MAPK1/3-mTOR and AKT-mTOR signalling pathways, and promotes apoptosis by repressing autophagy. Furthermore, SERINC5 interacting proteins IFITM1/2/3 not only regulate autophagy in a lysosomal-associated membrane protein LAMP1-dependent manner, but also activate the NF-κB signalling pathway by inhibiting autophagy after functional silencing. Interestingly, silencing IFITM1/2/3 enhances the inhibitory effect of SERINC5 on autophagy. These findings not only enrich the literature on SERINC5 and its interacting proteins IFITM1/2/3 in anti-CSFV, but also provide new ideas for controlling classical swine fever.

## Materials and methods

### Virus infection and biochemical intervention

PK-15 and 3D4/2 cells were cultured for 24 h before CSFV infection. 0.1 MOI CSFV or the same amount of medium with free serum were used to infect cells when cells had grown to approximately 80% confluence. Infection was followed by culture in a 5% CO_2_ incubator at 37 °C for 1.5 h, and then, cells were washed twice with PBS (pH 7.4). After that, cells were cultured in maintenance medium at 37 °C and 5% CO_2_ for 24 h or 48 h, after which they were harvested. For the biochemical intervention assay, cells were preincubated with the autophagy activator Rapamycin (Rapa) (100 nM) or the autophagy inhibitor 3-methyladenine (3-MA) (5 mM) at 37 °C and 5% CO_2_ for 1 h or 4 h, respectively, and then infected with (0.1 MOI) CSFV for 1.5 h. The same amount of DMSO was used as control. After cells had been cultured in fresh maintenance medium at 5% CO_2_ and 37 °C for 24 h, cell samples of each group were collected for further experiments.

### Virus titration

Virus titres of each treatment were obtained by 50% tissue culture infectious dose (TCID_50_) assays, which were measured according to a previous study [18]. In brief, cells were grown on 96-well plates and were inoculated with CSFV consisting of a 10-fold dilution series (10^−1^–10^−10^) for 2 days at 37 °C. The same amount of medium without CSFV was used as negative control and each dilution was repeated eight times. Cell culture supernatants were discarded. Cells were subsequently treated with absolute ethanol for 30 min at −20 °C. Then, cells were treated with 5% skim milk for 1.5 h. After that, the cells were incubated with mouse anti-CSFV E2 antibody (1:200) and Alexa Fluor488-labelled goat anti-mouse IgG (H+L) secondary antibody (1:200). Viral titres were measured by the Reed-Muench method.

### Plasmid construction and transfection

Full-length swine SERINC5 and IFITM1/2/3 were cloned using conventional PCR. Clones were inserted into the p3 × Flag-CMV and HA-CMV vector to generate p3 × Flag-SERINC5 and HA-IFITM1/2/3, respectively. The primer sequences used for SERINC5 and IFITM1/2/3 are presented in Table S1. Other plasmids, including mRFP/GFP-LC3, IFNβ-luc, NF-κB-luc, and pRL-TK were kept in the laboratory. The sequences of siRNAs targeting swine SERINC5, IFITM1/2/3, and LAMP1, as well as siNC are presented in Table S1. The plasmids and siRNAs were transfected into PK-15 and 3D4/2 cells, and were grown in 12-well plates at a density of 60–70%. Then, cells were transiently transfected with transfection mixture containing 1.5 µg plasmids (or 50 nM siRNA) and 2 μL lipofectamine 3000 (Thermo Fisher, L3000015) per well. At 4–6 h post transfection, cells were cultured in DMEM supplemented containing 2% FBS for 24 h, followed by infection with CSFV or biochemical intervention. The efficiency of gene and protein expression was tested by qRT-PCR and Western blotting.

### Co-Immunoprecipitation (Co-IP) and Western blotting

To immunoprecipitate SERINC5 in cell lysates, HEK-293T cells were transfected with HA-IFITM1/2/3 and Flag-SERINC5 for 24 h, followed by centrifugation. The supernatant was extracted and 40 µL protein A + G agarose beads were added. The mixture was immunoprecipitated with 1 µg anti-Flag mAb (Beyotime, AF519) overnight, followed by washing with PBS (pH 7.4) and collection for Western blotting with rabbit anti-HA mAb (Beyotime, AF2305).

Proteins were extracted by lysis on ice and were measured by Western blotting as previously reported [36]. The following antibodies were used: rabbit anti-SERINC5 (Abcam, ab204400); rabbit anti-Caspase3 (Beyotime, AC030); rabbit anti-PARP (Beyotime, AP102); rabbit anti-Caspase9 (Beyotime, AC062); rabbit anti-p-IκBA (Beyotime, AF5851); rabbit anti-P65 (Beyotime, AF0246); rabbit anti-p-mTOR (Abcam, ab109268); rabbit anti-CaMKII (Abcam, ab168818); mouse anti-IκBα (CST, 9247); rabbit anti-p-PRKAA/AMPKα (CST, 2535); rabbit anti-MAPK1/3 (CST, 9102); rabbit anti-p-MAPK1/3 (CST, 9101); mouse anti-IκB-Ras (Santa Cruz, sc -374,311); rabbit anti-Atg5 (CST, D5F5 U); rabbit anti-SQSTM1/p62 (CST, 3495); rabbit anti-BECN1 (CST, 3495); rabbit anti-LC3B (CST, 2775); rabbit anti-p-AKT (CST, 9271); rabbit anti-AKT (CST, 9272); rabbit anti-IFITM1 polyclonal antibody (CST, 13126); rabbit anti-IFITM2/3 (CST, 13530). GAPDH (Beyotime, AG019) or Tubulin (Beyotime, AT819) were used as loading controls.

### Yeast two-hybrid (Y2H) assay

Y2H assay was conducted according to our previous study [36]. In brief, the coding sequences of IFITM1/2/3 and SERINC5 were incorporated into prey pGADT7 and bait pGBKT7 vectors, respectively. IFITM1/2/3 and SERINC5 were co-transformed into the Y2H yeast strain to confirm their interaction. Primer sequences are listed in Table S1.

### Confocal fluorescence microscopy

According to requirements, cells were cultured to 30% confluence and were chemically pretreated as indicated. When needed, cells were co-transfected with mRFP-GFP-LC3 and plasmid/siRNAs as indicated, followed by infection/non-infection with CSFV for 24 h. Cells were then washed, fixed, permeabilized and blocked according to our previous study [36]. Next, cells were incubated with appropriate primary antibodies (anti-Flag mAb, anti-E2 mAb, or anti-HA mAb) and the corresponding fluorescent secondary antibody. After rinsing three times with TBST, cells were stained with DAPI (Beyotime, C1002). Fluorescent signals were visualized with a confocal fluorescence microscope (Leica TCS SP2, Germany).

### Dual luciferase reporter (DLR) assay

HEK-293T cells were transfected with plasmid encoding HA-CMV and HA-IFITM1/2/3 or siRNAs targeting swine IFITM1/2/3. The promoter reporter plasmids IFNβ, pRL-TK, and NF-κB were also seeded using the transfection reagent Lipofectamine 3000. Cells were stimulated with Sev for a further 24 h, followed by collection and dual luciferase assays (Promega, E1910) to analyse luciferase activity using a Dual-Luciferase Assay Kit (Promega, Madison, WI, USA). Six biological replicates were performed.

### Statistical analysis

All data are shown as mean ± SD of three biological replicates by one-way ANOVA using SPSS 16.0 software. SigmaPlot 12.0 software was used to create graphs. Significance levels were tested * *P* < 0.05, ** *P* < 0.01, *** *P* < 0.001.

## Results

### SERINC5 inhibits autophagy induced by CSFV infection

**Figure 1.**
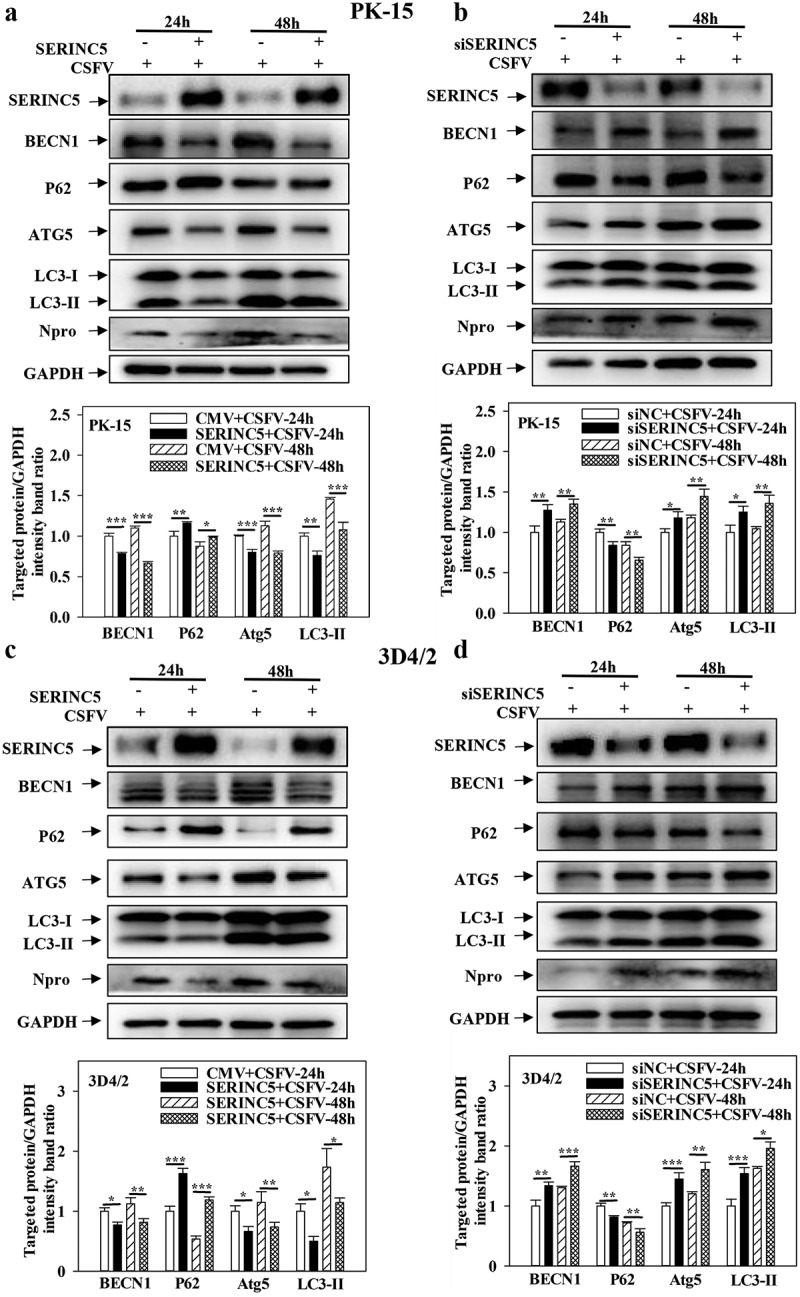
SERINC5 represses the expression level of autophagic proteins in CSFV infected cells. (a-d) The protein levels of BECN1, P62, LC3-I/II, ATG5, Npro and GAPDH were tested. PK-15 (a and b) and 3D4/2 (c and d) cells were transfected with 3 × Flag-SERINC5 or siSERINC5, then incubated with CSFV (MOI = 0.1) for 24 and 48 h. The level of proteins was carried out using Image-Pro Plus 6.0 software.

To identify the function of SERINC5 in the regulation of autophagy under CSFV infection, the levels of autophagy-related proteins LC3-I/II, P62, BECN1, and ATG5 were measured. As presented in Figure 1(a,c), overexpression of SERINC5 decreased LC3-I/II, BECN1, and ATG5 expressions, but increased P62 expression in CSFV-infected PK-15 and 3D4/2 cells. In contrast, knockdown of SERINC5 increased BECN1, LC3-I/II, and ATG5 expression, but decreased P62 expression in CSFV-infected PK-15 and 3D4/2 cells (Figure 1(b,d)). These data illustrated that SERINC5 represses autophagy in CSFV-infected cells.

Moreover, overexpression of SERINC5 inhibited autophagy in PK-15 and 3D4/2 cells treated with Rapa (Fig. S1A and S1C). SERINC5 knockdown promoted autophagy in these cells (Fig. S1B and S1D). These data further supported that SERINC5 represses autophagy.

**Figure 2.**
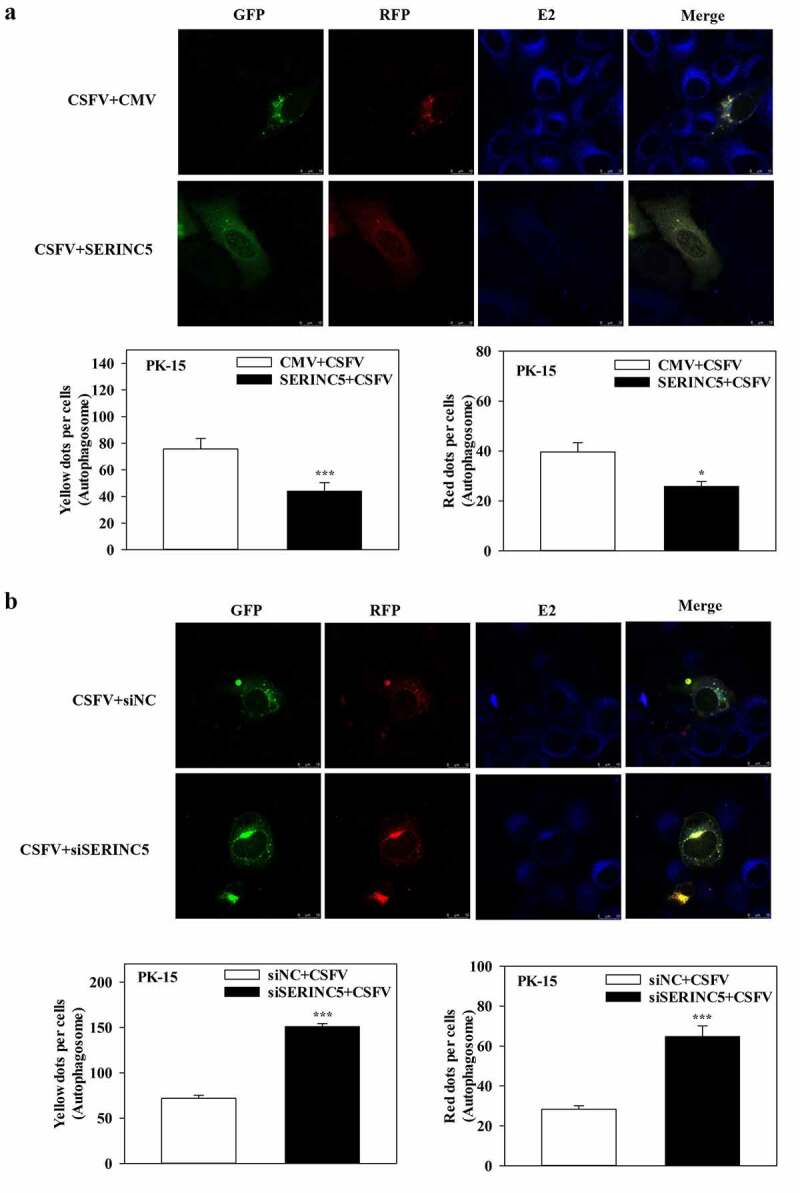
SERINC5 reduces the level of autophagic flux in CSFV infected PK-15 cells. (a and b) PK-15 cells transiently expressing mRFP-GFP-LC3 plasmid were transfected with 3 × Flag-SERINC5 (a) or siSERINC5 (b), and then incubated with CSFV (MOI = 0.1) for 24 h. Confocal fluorescence microscopy was used to capture the yellow dots (autophagosomes), red dots (autophagolysosomes) and blue spots (CSFV-E2 proteins). The GFP/RFP florescence intensity ratio was carried out using Image-Pro Plus 6.0 software. Scale bar: 10 µm.

Furthermore, to assess the effects of SERINC5 on the autophagy flux in CSFV-infected or Rapa-treated cells, confocal immunofluorescence microscopy was performed. The expression of autophagy dual-fluorescence reporter plasmid (mRFP-GFP-LC3) was measured. Overexpression of SERINC5 reduced autophagy flux, while knockdown of SERINC5 increased autophagy flux in CSFV-infected (Figure 2(a,b)) and Rapa-treated (Fig. S2A and S2B) PK-15 cells, as illustrated by the increase or decrease of the number of fluorescent dots. Red fluorescent dots indicate the formation of autophagolysosomes and increased autophagy flux (i.e. complete autophagy) in PK-15 cells. Yellow fluorescent dots (obtained by merging green and red fluorescence signals) indicate the formation of autophagosomes (i.e. incomplete autophagy). Collectively, these results corroborate the inhibitory role of SERINC5 in CSFV-induced complete autophagy.

### SERINC5 inhibits autophagy by MAPK1/3-mTOR and AKT-mTOR pathways

**Figure 3.**
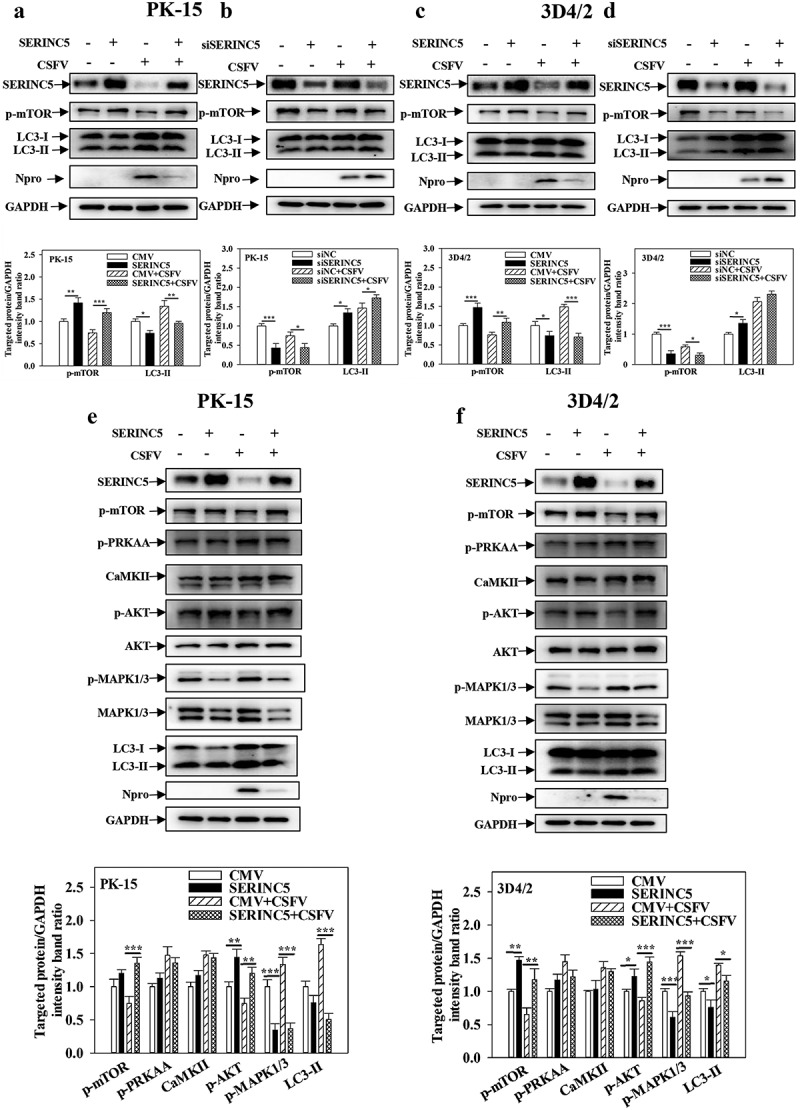
SERINC5 inhibits autophagy by AKT-mTOR and MAPK1/3-mTOR pathways in CSFV infected cells. (a-d) The protein levels of LC3-I/II, p-mTOR, Npro and GAPDH were assayed. PK-15 (a and b) and 3D4/2 (c and d) cells were transfected with 3 × Flag-SERINC5 or siSERINC5, and then were infected or uninfected with CSFV (MOI = 0.1) for 24 h. (e and f) The protein expression of p-mTOR, p-PRKAA, CaMKII, p-AKT, Akt, p-MAPK1/3, MAPK1/3, LC3-I/II, Npro and GAPDH were assayed PK-15 (e) and 3D4/2 (f) cells were transfected with 3 × Flag-SERINC5, and then were infected or uninfected with CSFV (MOI = 0.1) for 24 h. The level of proteins was carried out using Image-Pro Plus 6.0 software.

To determine the specific mechanism underlying the effect of SERINC5 on CSFV-induced autophagy, the protein levels of LC3-I/II and mTOR (proteins in mammalian target of the Rapa pathway) were measured, as these regulate the autophagy network in CSFV-infected cells [44]. Overexpression of SERINC5 enhanced mTOR phosphorylation (p-mTOR) expression and decreased LC3-I/II expression in CSFV-infected/uninfected PK-15 and 3D4/2 cells (Figure 3(a,c)). In contrast, knockdown of SERINC5 significantly reduced p-mTOR expression and enhanced LC3-I/II expression in CSFV-infected/uninfected PK-15 and 3D4/2 cells (Figure 3(b,d)). These results suggest that SERINC5 inhibits autophagy by activating mTOR signalling. To further test this suggestion, PK-15 and 3D4/2 cells were treated/untreated with Rapa. Then, the protein levels of mTOR and LC3-I/II were analysed in overexpressing or RNA-interfering SERINC5 cells. Similar results were obtained in Rapa-treated/untreated PK-15 and 3D4/2 cells (Fig. S3A-3D). These results further demonstrate that SERINC5 inhibits autophagy by activating mTOR signalling.

To further assess the function of SERINC5 in the mTOR signalling pathway in CSFV-infected cells, the protein levels of mTOR-related signalling pathways (i.e. CaMKII-PRKAA-mTOR, AKT-mTOR, and MAPK1/3-mTOR) were assessed by Western blotting. Overexpression of SERINC5 enhanced p-Akt expression and reduced p-MAPK1/3 expression in CSFV-infected/uninfected PK-15 and 3D4/2 cells, and also enhanced p-mTOR expression and decreased LC3-I/II expression. However, this treatment did not affect the expressions of p-PRKAA and CaMKII (Figure 3(e,f)). These results indicate that SERINC5 inhibits autophagy through MAPK1/3-mTOR and Akt-mTOR pathways.

### SERINC5 promotes apoptosis by inhibiting autophagy

**Figure 4.**
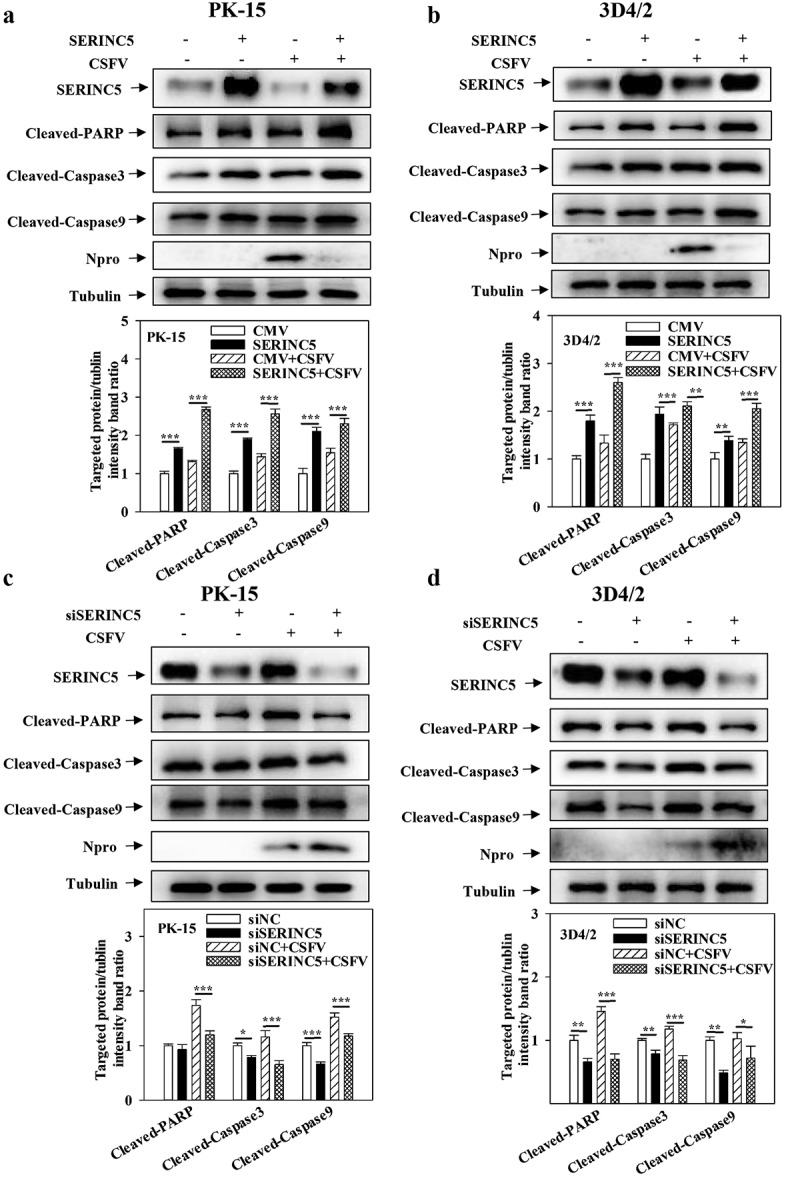
SERINC5 promotes apoptosis by inhibiting autophagy in CSFV infected cells. (a-d) The protein expression of Cleaved-PARP, Cleaved-Caspase3, Cleaved-Caspase9, Npro and Tubulin were assayed. PK-15 (a and c) and 3D4/2 (b and d) cells were transfected 3 × Flag-SERINC5 or siSERINC5, and then were infected or uninfected with CSFV (MOI = 0.1) for 24 h. The level of proteins was carried out using Image-Pro Plus 6.0 software.

To study the role of SERINC5 in apoptosis, the protein levels of apoptosis of Cleaved-Caspase3, Cleaved-Caspase9 and Cleaved-PARP were evaluated in SERINC5-overexpressing or -knockdown cells. The results indicate that the expressions of Cleaved-Caspase3, Cleaved-Caspase9, and Cleaved-PARP were enhanced in SERINC5-overexpressing cells (Figure 4(a,b)). However, their expressions decreased in RNA-interfering SERINC5 cells (Figure 4(c,d)), demonstrating that SERINC5 promotes apoptosis.

To further assess the different roles of SERINC5 between apoptosis and autophagy, the effects of overexpressing SERINC5 on apoptosis proteins in cells pre-treated with Rapa or 3-MA were measured. As presented in Fig. S4A and S4B, treatment with Rapa remarkably decreased the expressions of Cleaved-PARP, Cleaved-Caspase3, and Cleaved-Caspase9 expression in SERINC5-overexpressing PK-15 and 3D4/2 cells. However, treatment with 3-MA promoted Cleaved-Caspase3, Cleaved-Caspase9, and Cleaved-PARP expression in SERINC5-overexpressing PK-15 and 3D4/2 cells (Fig. S4C and S4D). The above data suggest that SERINC5 not only promotes apoptosis but achieves this by repressing autophagy.

### IFITM1/2/3 interacts with SERINC5 and inhibits CSFV replication

**Figure 5.**
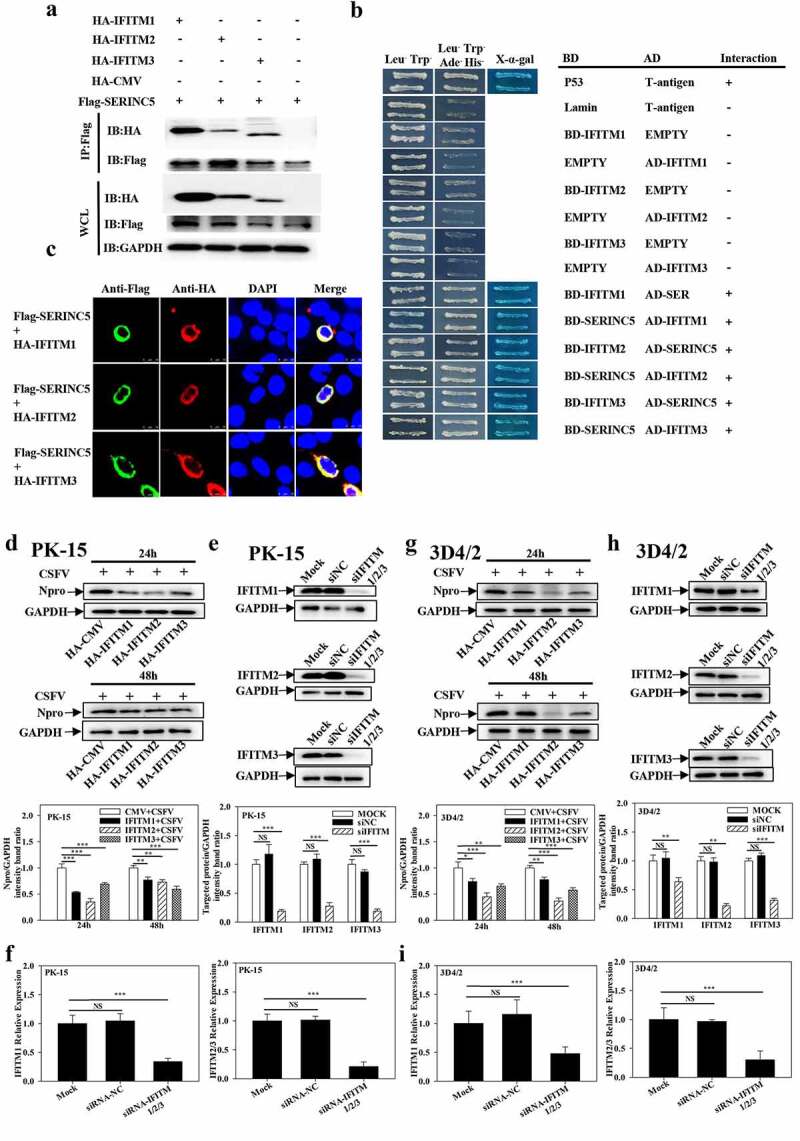
IFITM1/2/3 interacts with SERINC5 and effects CSFV replication. (a) HEK-293T cells were transfected with plasmids encoding Flag-SERINC5 and HA-IFITM1/2/3 protein, the cell lysates were collected and immunoprecipitated (IP) with anti-Flag mAb, and immunoblot (IB) analysis with anti-Flag mAb and anti-HA. (b) the interactions between IFITM1/2/3 and SERINC5 were analysed by Y2H. (c) Colocalization of IFITM1/2/3 and SERINC5. The yellow represents the co-localization of IFITM1/2/3 with SERINC5 protein. Scale bar; 10 µm. PK-15 (d) and 3D4/2 (g) cells were transfected with HA-IFITM1/2/3, and then were incubated with CSFV (MOI = 0.1) for 24 and 48 h. The protein levels of Npro and GAPDH were assayed. PK-15 (e, f) and 3D4/2 (h, i) cells were transfected with siNC or IFITM1/2/3 siRNA for 24 h. The siRNA silencing efficiency of IFITM1/2/3 were carried out using Western blotting (e, h) and qRT-PCR (f, i). The protein levels of Npro and GAPDH were assayed. The level of proteins was carried out using Image-Pro Plus 6.0 software.

Our previous study showed that SERINC5 may interact with IFITM1/2/3 by liquid chromatography-tandem mass spectrometry (LC-MS/MS) [36]. Herein, the interaction of IFITM1/2/3 with SERINC5 was validated using Co-IP, Y2H and colocalization assays (Figure 5(a–c)).

To assess the effect of IFITM1/2/3 on CSFV infection, the expression level of Npro was assessed using Western blotting in IFITM1/2/3-overexpressing cells at 24 h and 48 h post CSFV infection. Npro expression significantly decreased in IFITM1/2/3-overexpressing PK-15 and 3D4/2 cells (Figure 5(d,g). To explore the anti-CSFV effects of IFITM1/2/3, specific siRNAs were used to target IFITM1/2/3 in PK-15 and 3D4/2 cells. These siRNAs resulted in the knockdown of protein (Figure 5(e,h)) and gene expression levels (Figure 5(f,i)). Then, the expression of CSFV Npro was assessed by Western blotting in IFITM1/2/3-knockdown cells at 24 h and 48 h post-infection with CSFV. Knockdown of IFITM1/2/3 expression resulted in an upregulation of CSFV Npro protein compared with expressions in siNC-treated PK-15 and 3D4/2 cells (Fig. S5A and S5B). Collectively, these data suggest that IFITM1/2/3 inhibits CSFV replication.

### IFITM1/2/3 promotes autophagy induced by CSFV infection

**Figure 6.**
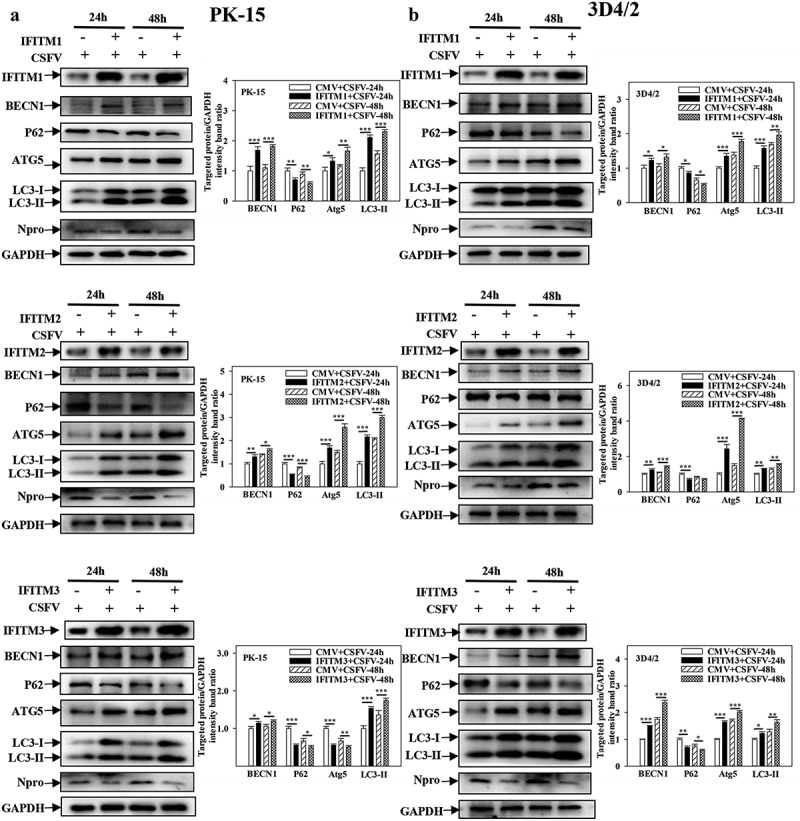
Overexpression of IFITM1/2/3 regulates the expression level of autophagic proteins in CSFV infected PK-15 and 3D4/2 cells. (a and b) The preotein levels of BECN1, P62, LC3-I/II, ATG5, Npro and GAPDH were assayed. PK-15 (a) and 3D4/2 (b) cells were transfected with HA-IFITM1/2/3 or siIFITM1/2/3, followed by incubated with CSFV (MOI = 0.1) for 24 and 48 h. The level of proteins was carried out using Image-Pro Plus 6.0 software.

**Figure 7.**
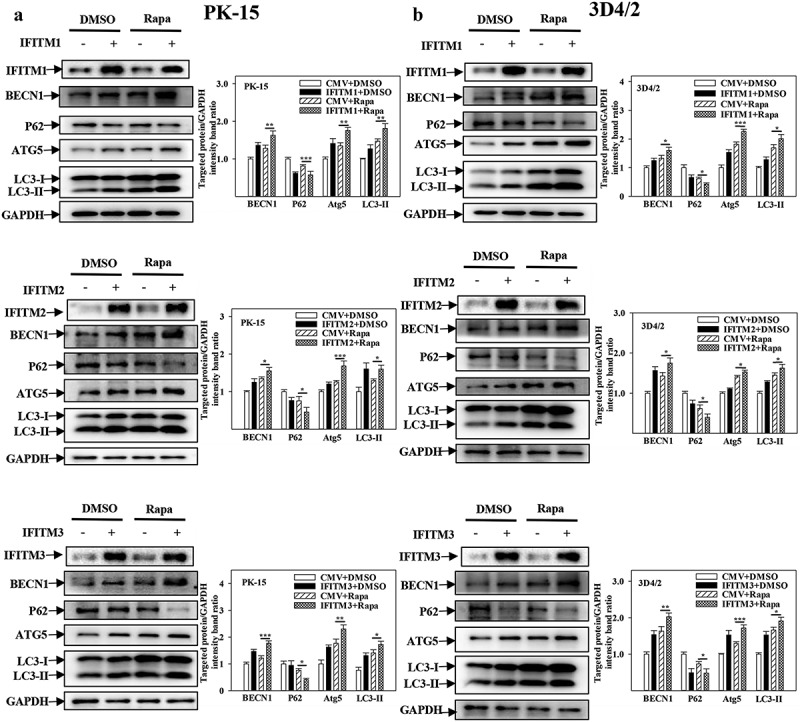
Overexpression of IFITM1/2/3 changes the expression level of autophagic proteins in Rapa treated PK-15 and 3D4/2 cells. (a and b) The protein levels of BECN1, P62, LC3-I/II, ATG5 and GAPDH were assayed. PK-15 (a) and 3D4/2 (b) cells were respectively pretreated with 100 nmol Rapa or equal amount of DMSO for 1 h, followed by transfected with HA-IFITM1/2/3 or siRNA of siIFITM1/2/3 for 24 h. The level of proteins was carried out using Image-Pro Plus 6.0 software.

IFITM1/2/3 interacts with SERINC5, which inhibits autophagy induced by CSFV infection. However, whether IFITM1/2/3 also regulates autophagy induced by CSFV infection remains unclear. To address this question, the effects of overexpression or knockdown of IFITM1/2/3 on the expressions of autophagic proteins in CSFV-infected PK-15 and 3D4/2 cells were assessed using Western blotting. The results indicate that overexpression of IFITM1/2/3 significantly induced BECN1, LC3-I/II, and ATG5 expression, as well as decreased p62 expression in CSFV-infected PK-15 and 3D4/2 cells (Figure 6(a,b)). In contrast, knockdown of IFITM1/2/3 decreased the protein expression of BECN1, LC3-I/II, and ATG5 as well as enhanced p62 expression in CSFV-infected PK-15 and 3D4/2 cells (Fig. S6A and S6B). These results show that overexpression of IFITM1/2/3 promotes autophagy, while knockdown of IFITM1/2/3 inhibits autophagy in CSFV-infected cells. In addition, overexpression or knockdown of IFITM1/2/3 promoted (Figure 7(a,b)) or inhibited (Fig. S7A and S7B) autophagy in Rapa-treated PK-15 and 3D4/2 cells, respectively. The results suggest that IFITM1/2/3 promotes autophagy.

**Figure 8.**
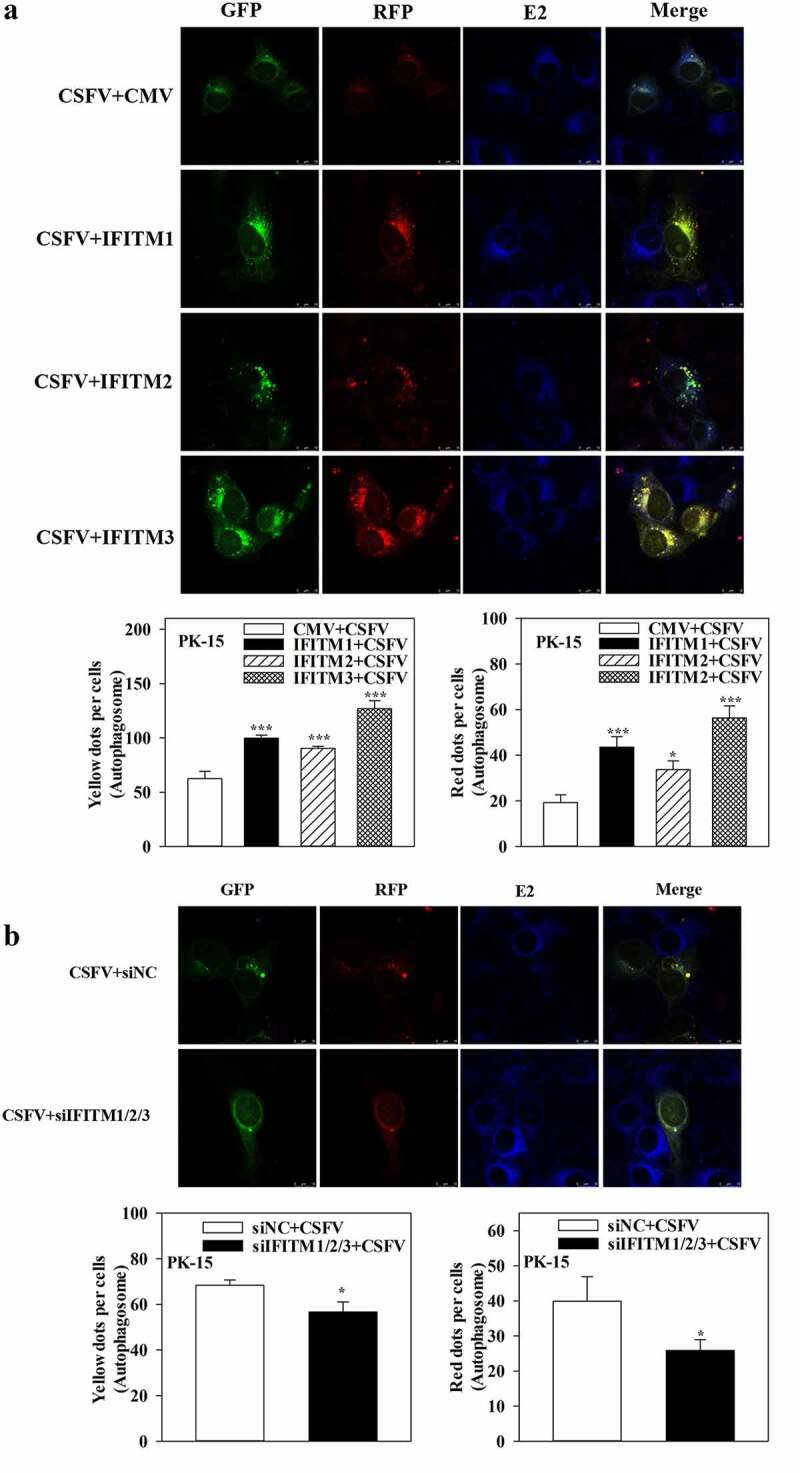
IFITM1/2/3 alter the level of autophagic flux in CSFV infected PK-15 cells. (a and b) PK-15 cells transiently expressing mRFP-GFP-LC3 plasmid were transfected with HA-IFITM1/2/3 (a) or siIFITM1/2/3 (b), and then incubated with CSFV (MOI = 0.1) for 24 h. Confocal fluorescence microscopy was used to capture the yellow dots (autophagosomes), red dots (autophagolysosomes) and blue spots (CSFV-E2 proteins). The GFP/RFP florescence intensity ratio were analysed using Image-Pro Plus 6.0 software. Scale bar: 10 µm.

Further research showed that overexpression of IFITM1/2/3 significantly increased autophagic flux in CSFV-infected (Figure 8(a)) or Rapa-treated (Fig. S8A) PK-15 cells; in contrast, knockdown of IFITM1/2/3 reduced autophagic flux in CSFV-infected (Figure 8(b)) or Rapa-treated (Fig. S8B) PK-15 cells. In summary, these results indicate the positive role of IFITM1/2/3 in CSFV-induced autophagy.

### IFITM1/2/3 regulates autophagy in a LAMP1-dependent manner

**Figure 9.**
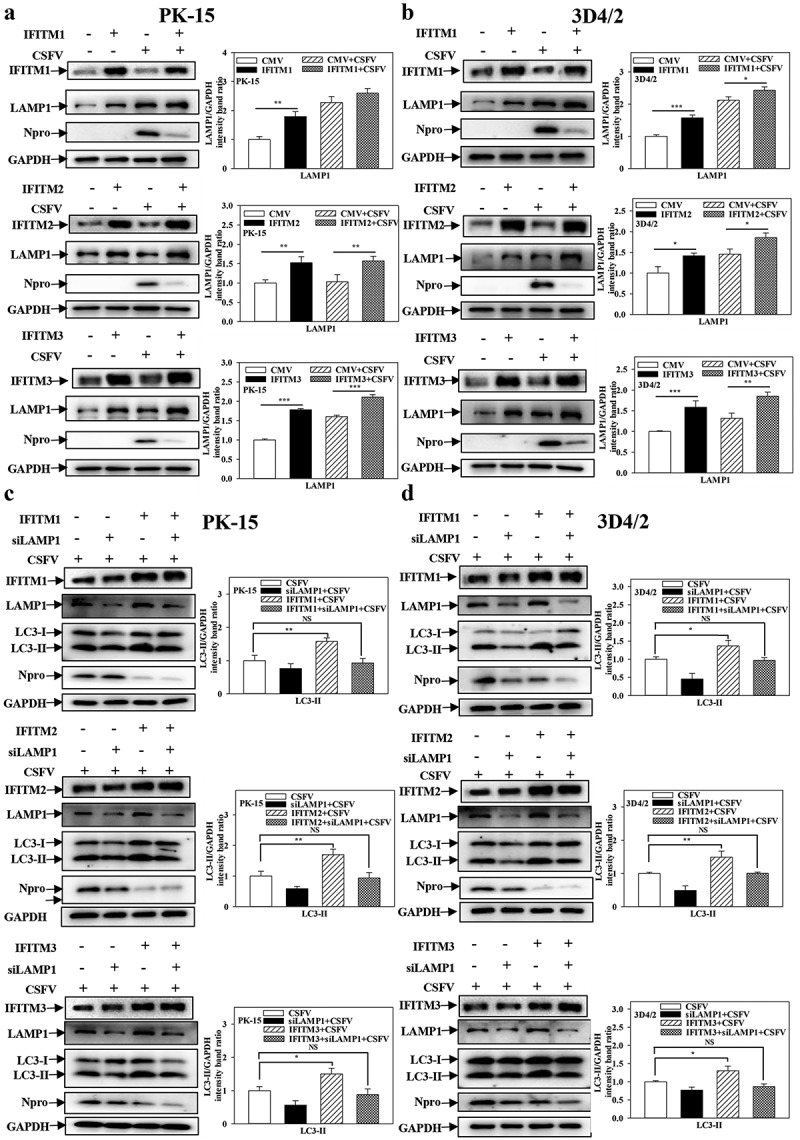
IFITM1/2/3 regulates autophagy in a LAMP1 protein dependent manner. (a and b) The protein levels of LAMP1, Npro and GAPDH were assayed. PK-15 (a) and 3D4/2 (b) cells were transfected with HA-IFITM1/2/3, and then were infected or uninfected with CSFV (MOI = 0.1) for 24 h. PK-15 (c) and 3D4/2 (d) cells were transfected with HA-IFITM1/2/3 and siLAMP1 for 24 h, followed by incubated with CSFV (MOI = 0.1) for another 24 h. The protein levels of LC3-I/II, LAMP1, Npro and GAPDH were assayed. The level of proteins was carried out using Image-Pro Plus 6.0 software.

Therefore, to further study the specific mechanism of IFITM1/2/3 of CSFV-induced autophagy, whether IFITM1/2/3 affects autophagy was explored by altering the expression of LAMP1, which is an evaluation criterion for lysosome function and co-localizes with IFITM1/2/3 in cells [42,45]. The protein level of LAMP1 was assessed using Western blotting. Overexpression of IFITM1/2/3 enhanced the level of LAMP1 expression (Figure 9(a,b)), while knockdown of IFITM1/2/3 significantly decreased the level of LAMP1 protein (Fig. S9A and S9B) in CSFV-infected/uninfected cells. The results indicate that IFITM1/2/3 affects lysosomal function by altering LAMP1 expression.

Furthermore, specific siRNAs were used to target LAMP1 in both PK-15 and 3D4/2 cells, which decreased the protein (Fig. S10A and S10B) and transcript (Fig. S10C and S10D) levels of LAMP1. The protein levels of LC3-I/II were measured in CSFV-infected PK-15 and 3D4/2 cells that were co-transfected with siLAMP1 and HA-IFITM1/2/3. Overexpression of IFITM1/2/3 markedly enhanced LC3-I/II expression. However, the expression levels of LC3-I/II were not significantly different when IFITM1/2/3 was overexpressed after interference of LAMP1 compared with a control group (Figure 9(c,d)). In summary, these results demonstrate that IFITM1/2/3 regulates autophagy in a lysosomal-associated membrane protein LAMP1-dependent manner.

### IFITM1/2/3 functional silencing activates the NF-κB signalling pathway by autophagy

**Figure 10.**
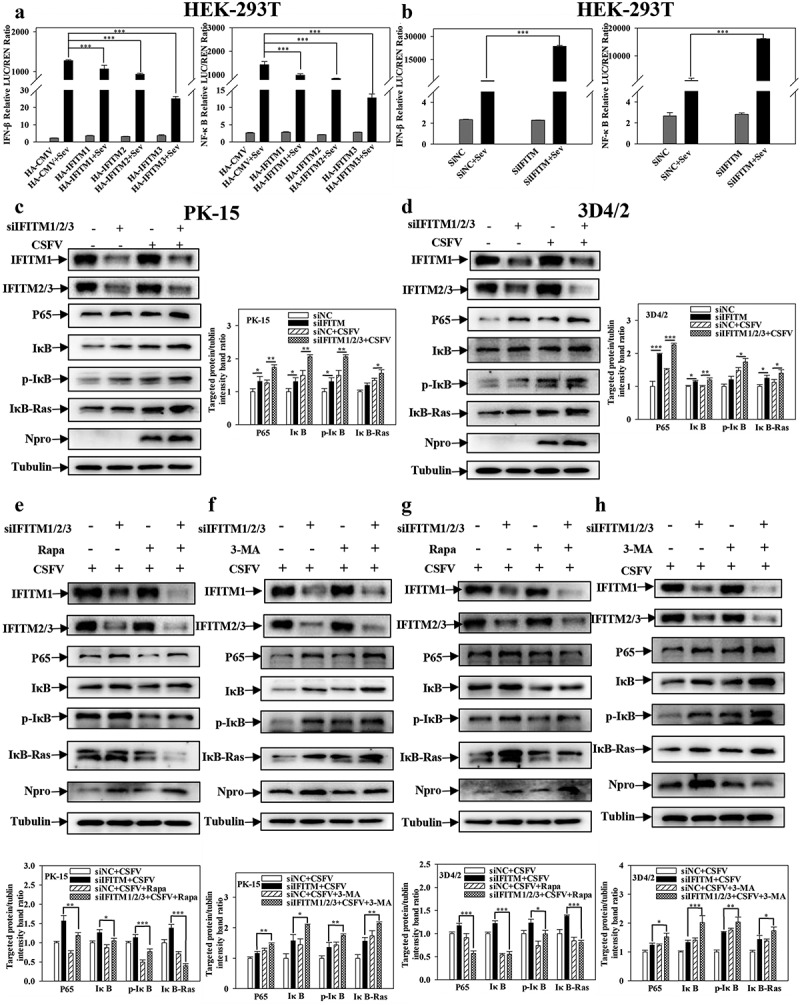
Pharmacological regulation of autophagic changes the regulation of IFITM1/2/3 on the NF-κB pathway. (a and b) Luciferase activities were tested by DLR..HEK-293T cells were transfected with HA-IFITM1/2/3 and pRL-TK (a) or siIFITM1/2/3 and pRL-TK (b) together with the NF-κB or IFNβ luciferase reporter for 24 h, followed by incubated with Sev for another 24 h. pRL-TK was used as an internal control. PK-15 (c) and 3D4/2 (d) cells were transfected with siIFITM1/2/3, and then were infected or uninfected with CSFV (MOI = 0.1) for 24 h. The protein expression of P65, IκB, p-IκB, IκB-Ras, Npro and Tubulin were assayed by Western blotting analysis. (e-g) The expression levels of P65, IκB, p-IκB, IκB-Ras, Npro and Tubulin were carried out using Western blotting. PK-15 (e and f) and 3D4/2 (f and h) cells were respectively pretreated with 100 nmol Rapa or 5 mM 3-MA or equal amount of DMSO for 1 h, and then transfected with siIFITM1/2/3 for 24 h, followed by incubated with CSFV (MOI = 0.1) for another 24 h. The level of proteins was carried out using Image-Pro Plus 6.0 software.

The NF-κB pathway is involved in CSFV infection, and IFITM3 may negatively regulate the transcription of NF-κB, induced by Sev [43]. To determine whether IFITM1/2/3 is associated with the NF-κB pathway in CSFV infection, the promoter activities of NF-κB-luc and IFNβ-luc were tested by DLR assay. Overexpression of IFITM1/2/3 significantly decreased the promoter activities of NF-κB-luc and IFNβ-luc in Sev-infected cells (Figure 10(a)). In contrast, knockdown of IFITM1/2/3 increased the promoter activities of NF-κB-luc and IFNβ-luc in Sev-infected cells (Figure 10(b)). Furthermore, Western blotting showed that knockdown of IFITM1/2/3 increased the expression levels of NF-κB pathway proteins p-IκB, IκB, IκB-Ras, and P65 in CSFV-infected PK-15 and 3D4/2 cells (Figure 10(c,d)). Likewise, the transcript levels of *IFNα*, *IFNβ*, and *TNFα* were increased in CSFV-infected PK-15 and 3D4/2 cells transfected with siIFITM1/2/3 (Fig. S11A and S11B). These results suggest that knockdown of IFITM1/2/3 up-regulates the NF-κB signalling pathway.

A previous study reported that the activation of the NF-κB signalling pathway is affected by autophagy [46], and that IFITM1/2/3 promotes autophagy. Furthermore, it was assessed whether the facilitation of the NF-κB signalling pathway by IFITM1/2/3 functional silencing is also related to autophagy. For this purpose, the effects of IFITM1/2/3 functional silencing on NF-κB pathway proteins in Rapa or 3-MA pre-treated cells were assessed. As shown in Figures 10(e,g), Rapa treatment remarkably decreased the levels of p-IκB, IκB, IκB-Ras and P65 in IFITM1/2/3 functional silenced cells. However, 3-MA treatment promoted the levels of P65, IκB, p-IκB, and IκB-Ras in IFITM1/2/3 functional silenced cells (Figure 10(f,h)). In summary, these results suggest that IFITM1/2/3 functional silencing regulates the NF-κB signalling pathway by repressing autophagy.

### IFITM1/2/3 functional silencing enhances the inhibition of autophagy by SERINC5

**Figure 11.**
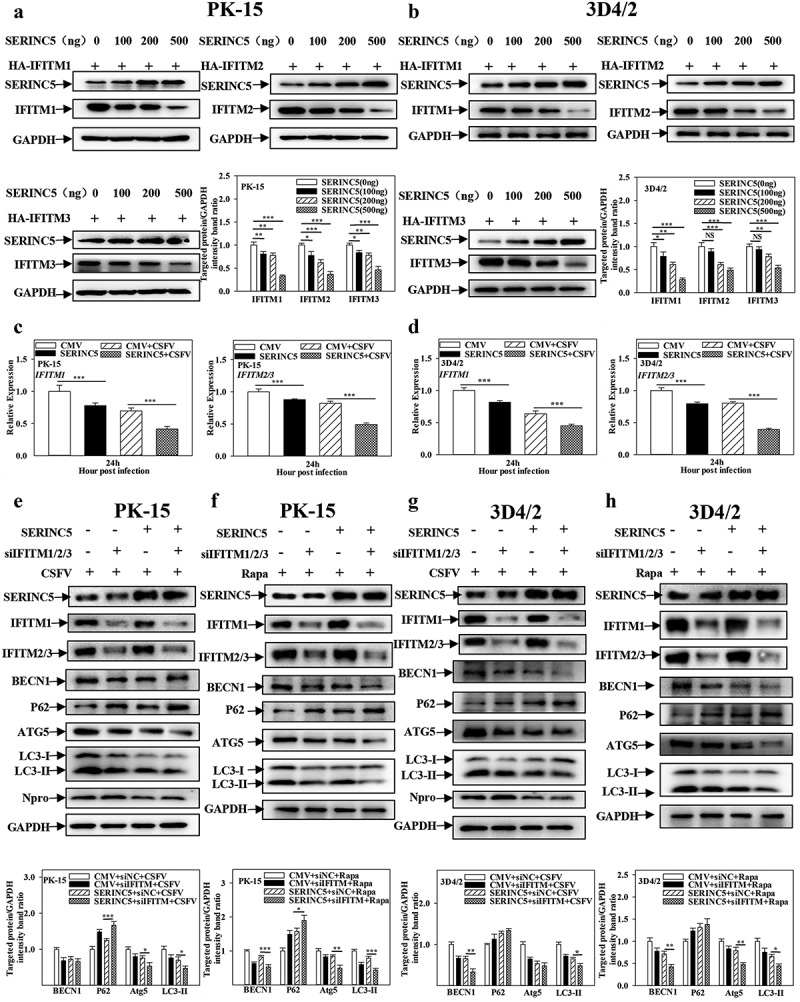
Interference of IFITM1/2/3 enhances the inhibitory effect of SERINC5 on autophagy pathway. (a and b) The protein levels of SERINC5, HA-IFITM1/2/3 and GAPDH were carried out using Western blotting. PK-15 (a) and 3D4/2 (b) cells were co-transfected with HA-IFITM1/2/3 and Flag-SERINC5 (0, 100, 200, 500 ng) for 24 h. (c and d) The transcription level of IFITM1/2/3 were analyzed by RT-qPCR. PK-15 (c) and 3D4/2 (d) cells were transfected with Flag-SERINC5, and then were infected or uninfected with CSFV (MOI = 0.1) for 24 h. (e-h) The protein levels of BECN1, P62, LC3-I/II, ATG5, Npro and GAPDH were assayed. PK-15 (e) and 3D4/2 (g) cells were transfected with siIFITM1/2/3 and 3 × Flag-SERINC5 for 24 h, followed by incubated with CSFV (MOI = 0.1) for another 24 h. PK-15 (f) and 3D4/2 (h) cells were respectively pretreated with 100 nmol Rapa, and then transfected with siIFITM1/2/3 for 24 h. The level of proteins was carried out using Image-Pro Plus 6.0 software.

The results presented above show that SERINC5 interacts with IFITM1/2/3, and both SERINC5 and IFITM1/2/3 can regulate CSFV-induced autophagy. Therefore, it was further studied whether the inhibitory effect of SERINC5 on autophagy is related to the interaction between SERINC5 and IFITM1/2/3. The effects of SERINC5 on the protein and transcript levels of IFITM1/2/3 were investigated using Western blotting and qRT-PCR assays in both PK-15 and 3D4/2 cells. SERINC5 decreased the protein (Figure 11(a,b)) and mRNA levels of IFITM1/2/3 (Figure 11(c,d)). Next, specific siRNA was used to inhibit IFITM1/2/3 and the effects of SERINC5 on autophagy protein expressions in CSFV-infected or Rapa-treated PK-15 and 3D4/2 cells were observed. Knockdown of IFITM1/2/3 significantly enhanced the inhibitory effect SERINC5 imposes on BECN1, ATG5, and LC3-I/II proteins, accompanied by an enhanced up-regulation effect of SERINC5 on P62 expression in CSFV infected (Figure 11(e,g)) or Rapa-treated (Figure 11(f,h)) PK-15 and 3D4/2 cells. In summary, these results indicate that knockdown of IFITM1/2/3 enhances autophagy inhibition by SERINC5.

## Discussion

Research on the relationship between the pathogenic action of CSFV and autophagy has always been a hot topic. Autophagy is an innate immune mechanism, which not only protects cells from invasion by pathogenic microorganisms, but also maintains cell homoeostasis [47]. Many studies have suggested that complete autophagy is needed for the efficient infection and proliferation of viruses (including CSFV) [18,48,49]. Our previous work indicated that CSFV infection induces autophagy and increases virion release from host cells [18,19]. The cellular proteins of the host have also developed a variety of ways to resist CSFV infection and virus replication, in which autophagy is centrally important.

The transmembrane protein SERINC5 has been verified to play an important role in the replication of many viruses [34,35], and SERINC5 has been shown to inhibit CSFV replication [36]. However, the specific action of SERINC5 in the interaction between CSFV infection and host cells has not been sufficiently characterized. Moreover, the relationship between SERINC5 and autophagy has not been reported to date. Herein, it was found that SERINC5 inhibits autophagy (Figure 1 and Fig. S1), and that SERINC5 blocks autophagic flux and inhibits complete autophagy in CSFV-infected or Rapa-treated cells (Figure 2 and Fig. S2).

In the process of autophagy, mTOR is a key negative regulator of autophagy, and mTOR expression is suppressed in starvation-stimulated induced autophagy [50,51]. Our previous work reported that CSFV induces autophagy via the CaMKII-PRKAA-mTOR, MAPK1/3-mTOR, and AKT-mTOR signalling pathways [22]. To determine the underlying mechanism with which SERINC5 modulates CSFV-induced autophagy, the effect of SERINC5 on the mTOR pathway was assessed in CSFV-infected cells. The results showed that SERINC5 overexpression reduced LC3-II expression but increased p-mTOR expression, while SERINC5 knockdown results in the opposite (Figure 3(a–d) and Fig. S3). Furthermore, it has been reported that CSFV-infected cells reduced p-AKT expression, while the protein levels of p-MAPK1/3, p-PRKAA, and CaMKII were increased; thus, the activity of mTOR was inhibited and autophagy was induced [22]. The present study provides evidence demonstrating that SERINC5 overexpression increases the level of p-Akt and reduces the p-MAPK1/3, but does not affect the expression levels of p-PRKAA and CaMKII (Figure 3(e,f)). This result suggests that SERINC5 inhibits autophagy via MAPK1/3-mTOR and Akt-mTOR pathways.

Apoptosis induced by virus infecting is an important mechanism of virus pathogenicity, and is regarded as the first line of defence host cells employ against virus infection [52,53]. Our previous research showed that CSFV-infection-induced autophagy represses cell apoptosis, while shRNA-mediated autophagy inhibition promotes cell apoptosis [19]. To date, the function of SERINC5 in cell apoptosis remains unclear. The obtained results confirmed that SERINC5 overexpression increased Cleaved-PARP, Cleaved-Caspase3, and Cleaved-Caspase9 expression, whereas SERINC5 knockdown decreased their protein expressions (Figure 4(a–d)), indicating that SERINC5 promotes apoptosis. Additionally, this SERINC5-mediated promotion of apoptosis was more pronounced in 3-MA pre-treated cells, but it was remarkably decreased in Rapa pre-treated cells (Fig. S4). This suggests that SERINC5 increases the occurrence of apoptosis by inhibiting autophagy, thereby inhibiting viral replication.

Previously, IFITM protein candidates, which potentially interact with SERINC5, were identified by LC-MS [36]. The present study further verified the interaction between IFITM1/2/3 and SERINC5 by Y2H, Co-IP, and subcellular co-localization (Figure 5(a–c)). Additionally, IFITM1/2/3 overexpression was found to down-regulate the level of CSFV Npro protein, whereas IFITM1/2/3 knockdown promoted CSFV replication (Figure 5(d,g)), and Fig. S5A and S5B). These findings identify IFITM1/2/3 as an anti-CSFV protein, which is consistent with previous findings [42]. A previous study found that IFITM3 induced autophagy in influenza virus infection [54], further showing that IFITM1/2/3 also induced autophagy in CSFV-infected or Rapa-treated cells (Figure 6–8 and Figs. S6–8).

The autophagy process is commonly divided into the early stage of autophagosome formation and the late stage of autophagosome fusion with the lysosome thus forming the autophagolysosome, which is accompanied by the degradation of cargo proteins [55,56]. Importantly, lysosomal activity is a key factor in the degradation of cargo proteins [45]. Recent studies showed that lysosomal activity can be assessed by detecting lysosomal acidification (i.e. the lysosomal pH) and lysosomal function (i.e. the expression level of lysosome-associated membrane protein LAMP1) [57]. Furthermore, Li et al. (2020) found that IFITM1/2/3 co-localizes with LAMP1 [42]. The present work showed that IFITM1/2/3 was found to affect lysosomal function by altering LAMP1 protein expression (Figure 9(a,b), and Fig. S9A and S9B). Additionally, the promotion of autophagy by IFITM1/2/3 protein disappeared when RNA targeted LAMP1 protein (Figure 9(c,d)), indicating that IFITM1/2/3 regulates autophagy in a lysosomal-associated membrane protein LAMP1-dependent manner.

NF-κB activation has been assumed to be one of the crucial pathogenic mechanisms. Several previous studies have reported that the NF-κB system can be exploited by viruses including the hepatitis C virus, respiratory syndrome virus (PRRSV), and CSFV [58–60]. IFITMs, a family of small transmembrane proteins, can be induced by interferons and viral infection [43]. Furthermore, it has been demonstrated that IFITM3 negatively regulates the type I IFN pathway by mediating autophagic degradation of IRF3 and may also inhibit Sev-triggered induction of NF-κB [43]. Herein, the data showed that the NF-κB signalling pathway is activated by IFITM1/2/3 suppression (Figure 10(a–d) and Fig. S11A and S11B). Moreover, prior evidence suggested that both the NF-κB signalling pathway and IFITM3 are closely related to autophagy [43,46]. Further results indicated that knockdown of IFITM1/2/3 activates the NF-κB signalling pathway by inhibiting autophagy, thereby regulating immunoregulation (Figure 10(e–h)). Importantly, the obtained data showed that SERINC5 inhibits IFITM1/2/3 expression, and knockdown of IFITM1/2/3 enhanced the inhibitory effect of SERINC5 on autophagy (Figure 11). Combined with the above study, SERINC5 inhibits autophagy, while IFITM1/2/3 promotes autophagy. This indicates that SERINC5 and IFITM1/2/3 may have a dynamic balancing function in regulating autophagy. Nevertheless, the specific regulatory mechanism underlying this dynamic balance requires further clarification.

In conclusion, the current study demonstrated for the first time that SERINC5 inhibits autophagy by activating both the AKT-mTOR and MAPK1/3-mTOR signalling pathways, thereby promoting apoptosis. IFITM1/2/3 was found to interact with SERINC5 and regulate autophagy by affecting lysosomal function in a lysosomal-associated membrane protein LAMP1-dependent manner. Additionally, IFITM1/2/3 functional silencing not only promotes cytokine transcription by inhibiting autophagy, thus activating the NF-κB signalling pathway, but also enhances the inhibitory effect SERINC5 imposes on autophagy. These results on the action of SERINC5 and its interaction protein IFITM1/2/3 in CSFV-infected cells provide a scientific basis for the control and prevention of CSFV infection, suggesting potential targets for antiviral drugs.

### Abbreviations

3D4/2: Porcine alveolar macrophage
3-MA: 3-methyladenine
AD: Activation domain
AKT: AKT serine/threonine kinase
AMPK: Adenosine monophosphate-activated protein kinase
ATG5: Autophgy related 5
BD: Binding domain
BECN1: Beclin 1
CaMKII: Calcium/calmodulin dependent protein kinase kinase 2
CSFV: Classical swine fever virus
Co-IP: Co-immunoprecipitation
DLR: Dual luciferase reporter
DMSO: Dimethyl sulphoxide
GFP: Green fluorescent protein
IFITM: Interferon-induced transmembrane protein
IFN: Interferon
ISG: IFN-stimulated gene
LAMP1: Lysosomal-associated membrane protein
LC-MS: Liquid chromatography-mass spectrometry
MAP1LC3/LC3: Microtubule associated protein 1 light chain 3
MAPK: Mitogen-activated protein kinase
MOI: Multiplicity of infection
mTOR: Mechanistic target of rapamycin kinase
NF-κB: Nuclear factor kappa B subunit 1
PBS: Phosphate buffered saline
PK-15: Porcine kidney cells
PRKAA: Protein kinase AMP-activated catalytic subunit alpha
qRT-PCR: quantitative real-time PCR
Rapa: Rapamycin
RFP: Red fluorescent protein
SERINC5: Serine Incorporator 5
siRNA: Small interfering
SQSTM1/p62: Sequestosome 1
TBST: Tris buffered saline with tween solution
TNF: Tumor necrosis factor
Y2H: Yeast two-hybrid.

## Supplementary Material

Supplemental Material

## Data Availability

All data that support the findings of this study are available from the corresponding author upon reasonable request.
