# Supplementary material for: Interaction of SERINC5 and IFITM1/2/3 regulates the autophagy-apoptosis-immune network under CSFV infection
Source: Virulence. 2022 Oct 7;13(1):1720–40. doi: 10.1080/21505594.2022.2127241 (PMC9553151; doi:10.1080/21505594.2022.2127241)
Supplement: Supplemental Material [file KVIR_A_2127241_SM5799.pdf]

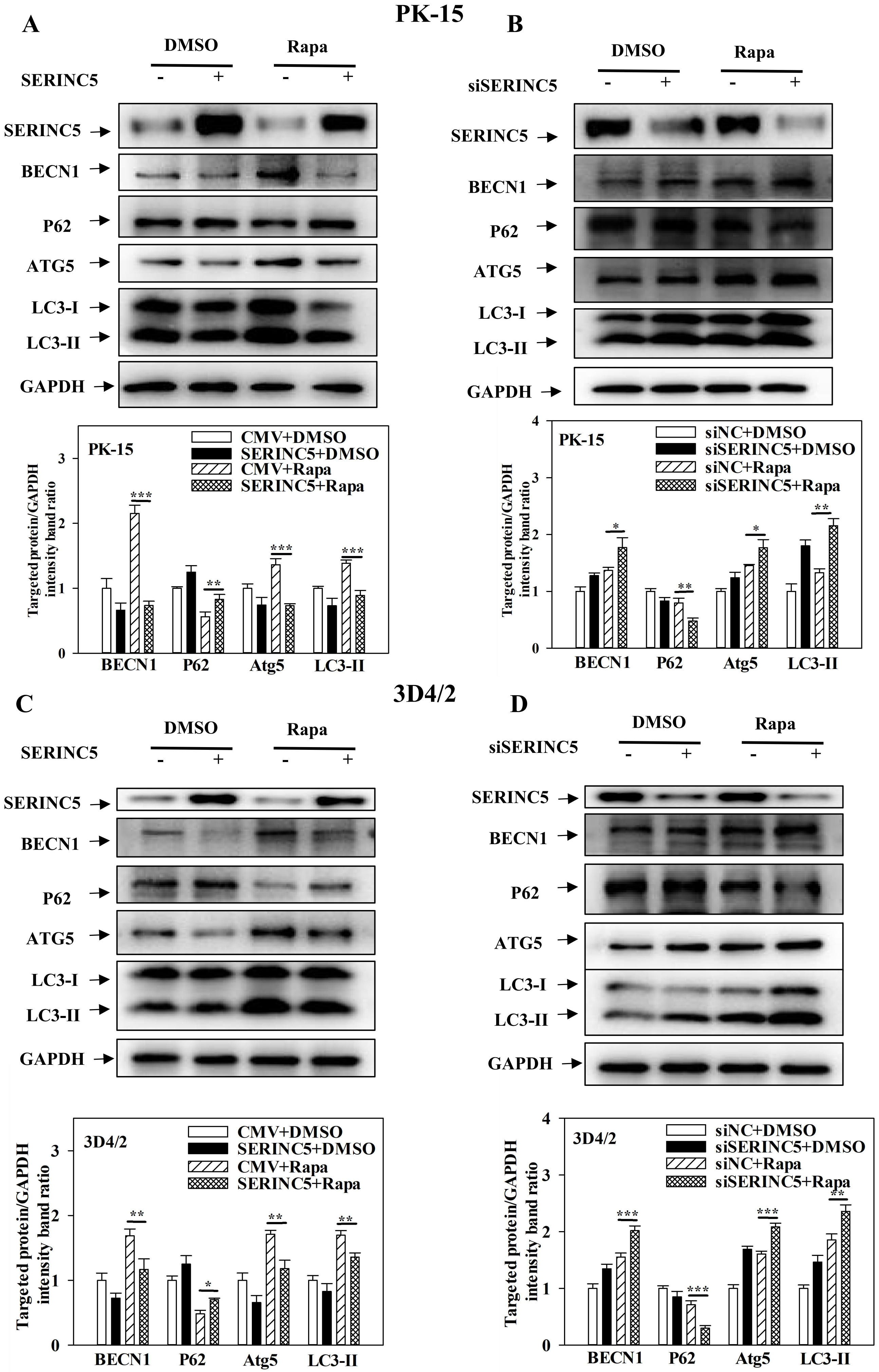
PK-15
A
DMSO
Rapa
SERINC5
- + - +
SERINC5
BECN1
P62
ATG5
LC3-I
LC3-II
GAPDH
PK-15
CMV+DMSO
SERINC5+DMSO
CMV+Rapa
SERINC5+Rapa
Targeted protein/GAPDH intensity band ratio
0
1
2
3
BECN1
P62
Atg5
LC3-II
B
DMSO
Rapa
siSERINC5
- + - +
SERINC5
BECN1
P62
ATG5
LC3-I
LC3-II
GAPDH
PK-15
siNC+DMSO
siSERINC5+DMSO
siNC+Rapa
siSERINC5+Rapa
Targeted protein/GAPDH intensity band ratio
0
1
2
3
4
BECN1
P62
Atg5
LC3-II
3D4/2
C
DMSO
Rapa
SERINC5
- + - +
SERINC5
BECN1
P62
ATG5
LC3-I
LC3-II
GAPDH
3D4/2
CMV+DMSO
SERINC5+DMSO
CMV+Rapa
SERINC5+Rapa
Targeted protein/GAPDH intensity band ratio
0
1
2
3
BECN1
P62
Atg5
LC3-II
D
DMSO
Rapa
siSERINC5
- + - +
SERINC5
BECN1
P62
ATG5
LC3-I
LC3-II
GAPDH
3D4/2
siNC+DMSO
siSERINC5+DMSO
siNC+Rapa
siSERINC5+Rapa
Targeted protein/GAPDH intensity band ratio
0
1
2
3
4
BECN1
P62
Atg5
LC3-II

Fig. S1 SERINC5 represses the expression level of autophagic proteins in Rapa treated cells. PK-15 **(A and B)** and 3D4/2 **(C and D)** cells were respectively pretreated with 100 nmol Rapa or equal amount of DMSO for 1 h, and then transfected with 3 × Flag-SERINC5 or siSERINC5 for 24 h. The level of proteins was carried out using Image-Pro Plus 6.0 software.

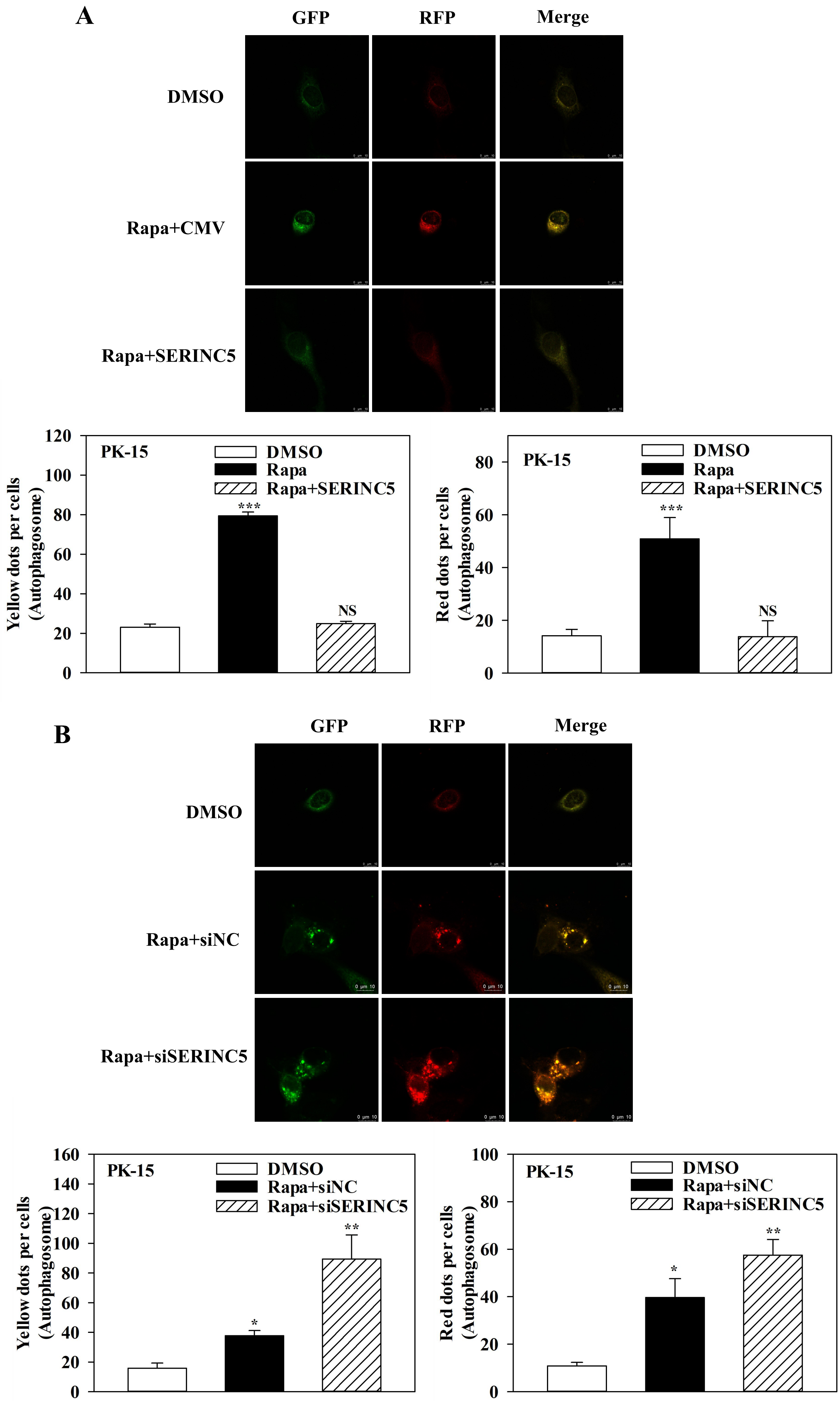
A
GFP
RFP
Merge
DMSO
Rapa+CMV
Rapa+SERINC5
PK-15
DMSO
Rapa
Rapa+SERINC5
***
NS
Yellow dots per cells (Autophagosome)
0
20
40
60
80
100
120
PK-15
DMSO
Rapa
Rapa+SERINC5
***
NS
Red dots per cells (Autophagosome)
0
20
40
60
80
B
GFP
RFP
Merge
DMSO
Rapa+siNC
Rapa+siSERINC5
PK-15
DMSO
Rapa+siNC
Rapa+siSERINC5
*
**
Yellow dots per cells (Autophagosome)
0
20
40
60
80
100
120
140
160
PK-15
DMSO
Rapa+siNC
Rapa+siSERINC5
*
**
Red dots per cells (Autophagosome)
0
20
40
60
80
100

Fig. S2 SERINC5 reduces the level of autophagic flux in Rapa treated PK-15 cells. **(A and B)** PK-15 cells were respectively pretreated with 100 nmol Rapa or equal amount of DMSO for 1 h, and then co-transfected mRFP-GFP-LC3 plasmid with 3 × Flag-SERINC5 **(A)** or siSERINC5 **(B)** for 24 h. Confocal fluorescence microscopy was used to capture the yellow dots (autophagosomes) and red dots (autophagolysosomes). The GFP/RFP florescence intensity ratio was carried out using Image-Pro Plus 6.0 software. Scale bar: 10 μm.

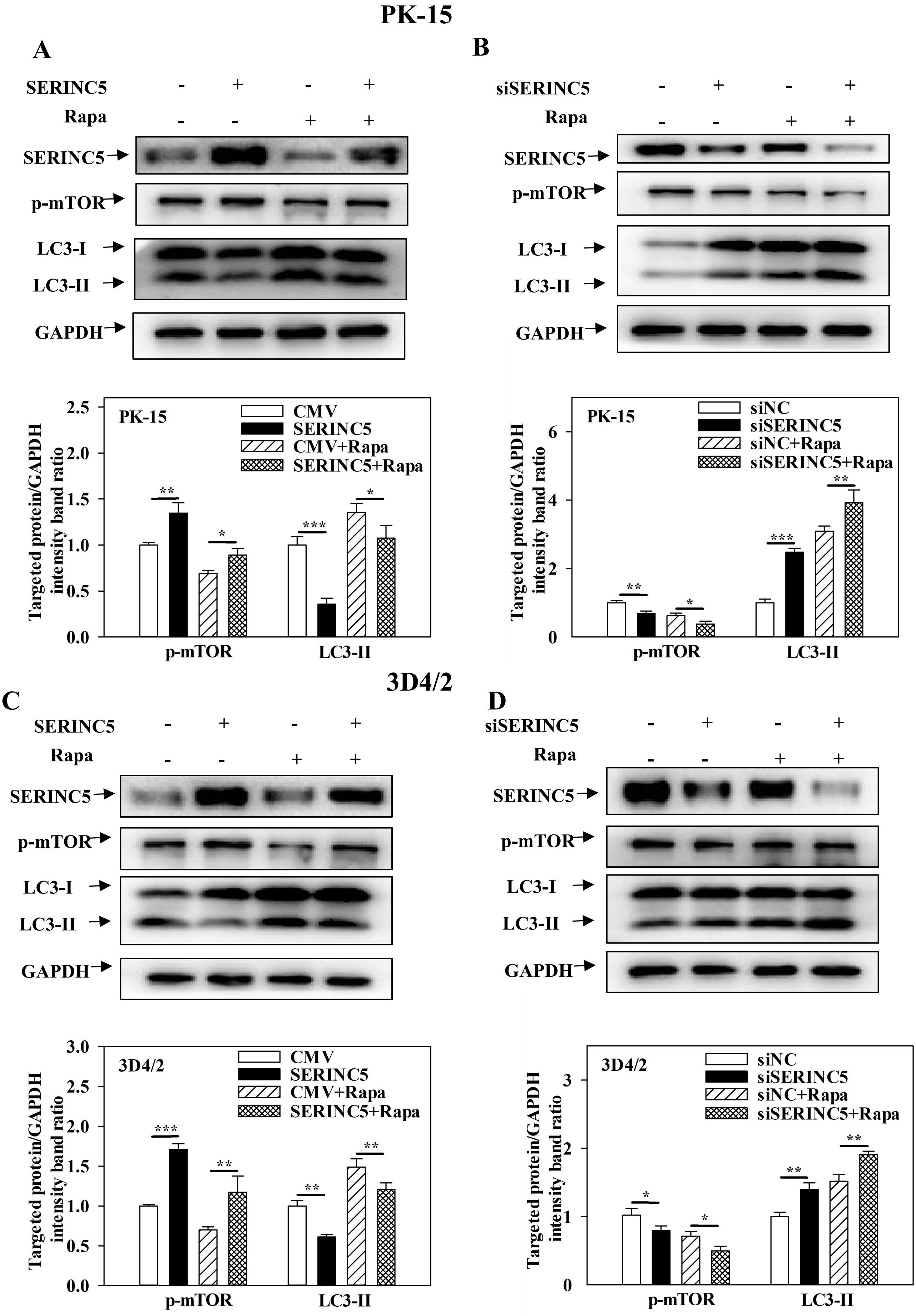
PK-15
A
SERINC5
Rapa
SERINC5
p-mTOR
LC3-I
LC3-II
GAPDH
PK-15
CMV
SERINC5
CMV+Rapa
SERINC5+Rapa
Targeted protein/GAPDH intensity band ratio
p-mTOR
LC3-II
B
siSERINC5
Rapa
SERINC5
p-mTOR
LC3-I
LC3-II
GAPDH
PK-15
siNC
siSERINC5
siNC+Rapa
siSERINC5+Rapa
Targeted protein/GAPDH intensity band ratio
p-mTOR
LC3-II
3D4/2
C
SERINC5
Rapa
SERINC5
p-mTOR
LC3-I
LC3-II
GAPDH
3D4/2
CMV
SERINC5
CMV+Rapa
SERINC5+Rapa
Targeted protein/GAPDH intensity band ratio
p-mTOR
LC3-II
D
siSERINC5
Rapa
SERINC5
p-mTOR
LC3-I
LC3-II
GAPDH
3D4/2
siNC
siSERINC5
siNC+Rapa
siSERINC5+Rapa
Targeted protein/GAPDH intensity band ratio
p-mTOR
LC3-II

Fig. S3 SERINC5 inhibits autophagy by AKT-mTOR and MAPK1/3-mTOR pathways in Rapa treated cells. **(A-D)** The protein levels of LC3-I/II, p-mTOR, Npro and GAPDH were assayed. PK-15 **(A and B)** and 3D4/2 **(C and D)** cells were respectively pretreated with 100 nmol Rapa or equal amount of DMSO for 1 h, and then transfected with 3 × Flag-SERINC5 or siSERINC5 for 24 h. The level of proteins was carried out using Image-Pro Plus 6.0 software.

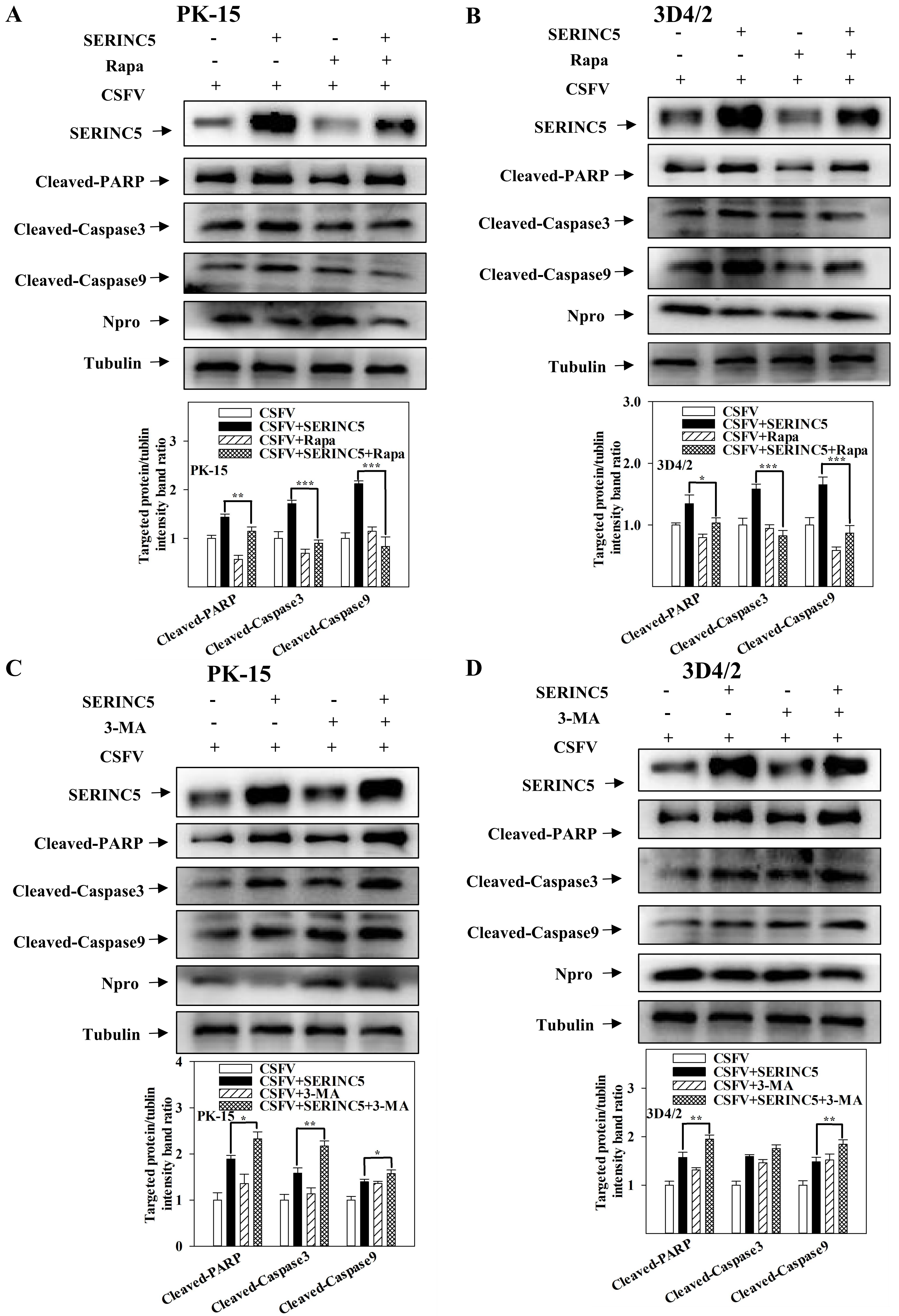
A
PK-15
SERINC5
Rapa
CSFV
SERINC5
Cleaved-PARP
Cleaved-Caspase3
Cleaved-Caspase9
Npro
Tubulin
CSFV
CSFV+SERINC5
CSFV+Rapa
CSFV+SERINC5+Rapa
Targeted protein/tublin intensity band ratio
PK-15
B
3D4/2
SERINC5
Rapa
CSFV
SERINC5
Cleaved-PARP
Cleaved-Caspase3
Cleaved-Caspase9
Npro
Tubulin
CSFV
CSFV+SERINC5
CSFV+Rapa
CSFV+SERINC5+Rapa
Targeted protein/tublin intensity band ratio
3D4/2
C
PK-15
SERINC5
3-MA
CSFV
SERINC5
Cleaved-PARP
Cleaved-Caspase3
Cleaved-Caspase9
Npro
Tubulin
CSFV
CSFV+SERINC5
CSFV+3-MA
CSFV+SERINC5+3-MA
Targeted protein/tublin intensity band ratio
PK-15
D
3D4/2
SERINC5
3-MA
CSFV
SERINC5
Cleaved-PARP
Cleaved-Caspase3
Cleaved-Caspase9
Npro
Tubulin
CSFV
CSFV+SERINC5
CSFV+3-MA
CSFV+SERINC5+3-MA
Targeted protein/tublin intensity band ratio
3D4/2

Fig. S4 SERINC5 promotes apoptosis by inhibiting autophagy in Rapa and 3-MA treated cells. **(A-D)** The protein expression of Cleaved-PARP, Cleaved-Caspase3, Cleaved-Caspase9, Npro and Tubulin were assayed. PK-15 **(A and C)** and 3D4/2 **(B and D)** cells were respectively pretreated with 100 nmol Rapa or 5 mM 3-MA or equal amount of DMSO for 1 h, and then transfected with 3 × Flag-SERINC5 for 24 h, followed by incubated with CSFV (MOI = 0.1) for another 24 h. The level of proteins was carried out using Image-Pro Plus 6.0 software.

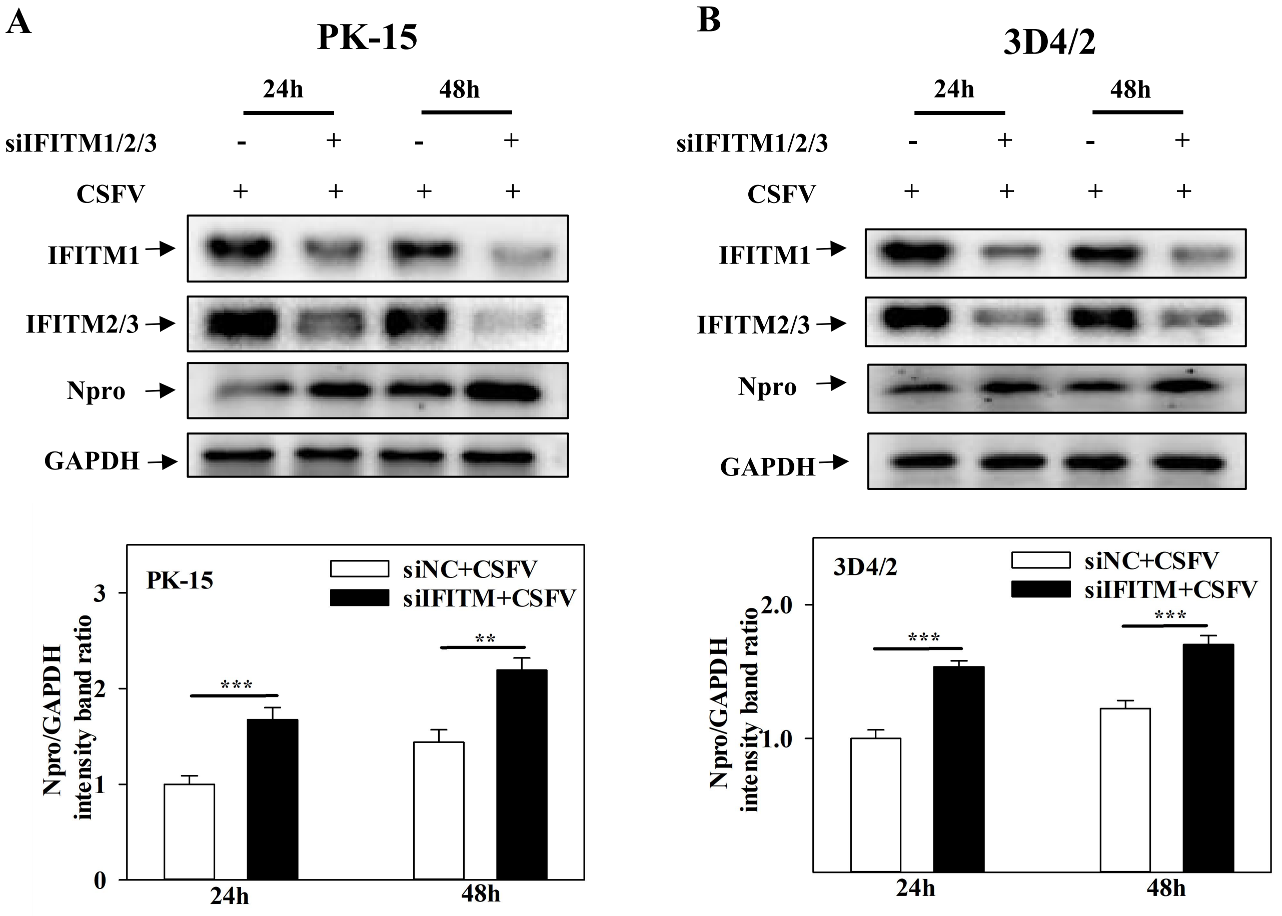

Fig. S5 Silencing of IFITM1/2/3 represses CSFV Npro protein expression. PK-15 **(A)** and 3D4/2 **(B)** cells were transfected with the siRNA of siIFITM1/2/3, then were incubated with CSFV (MOI = 0.1) for 24 and 48 h. The protein levels of Npro and GAPDH were assayed. The level of proteins was carried out using Image-Pro Plus 6.0 software.

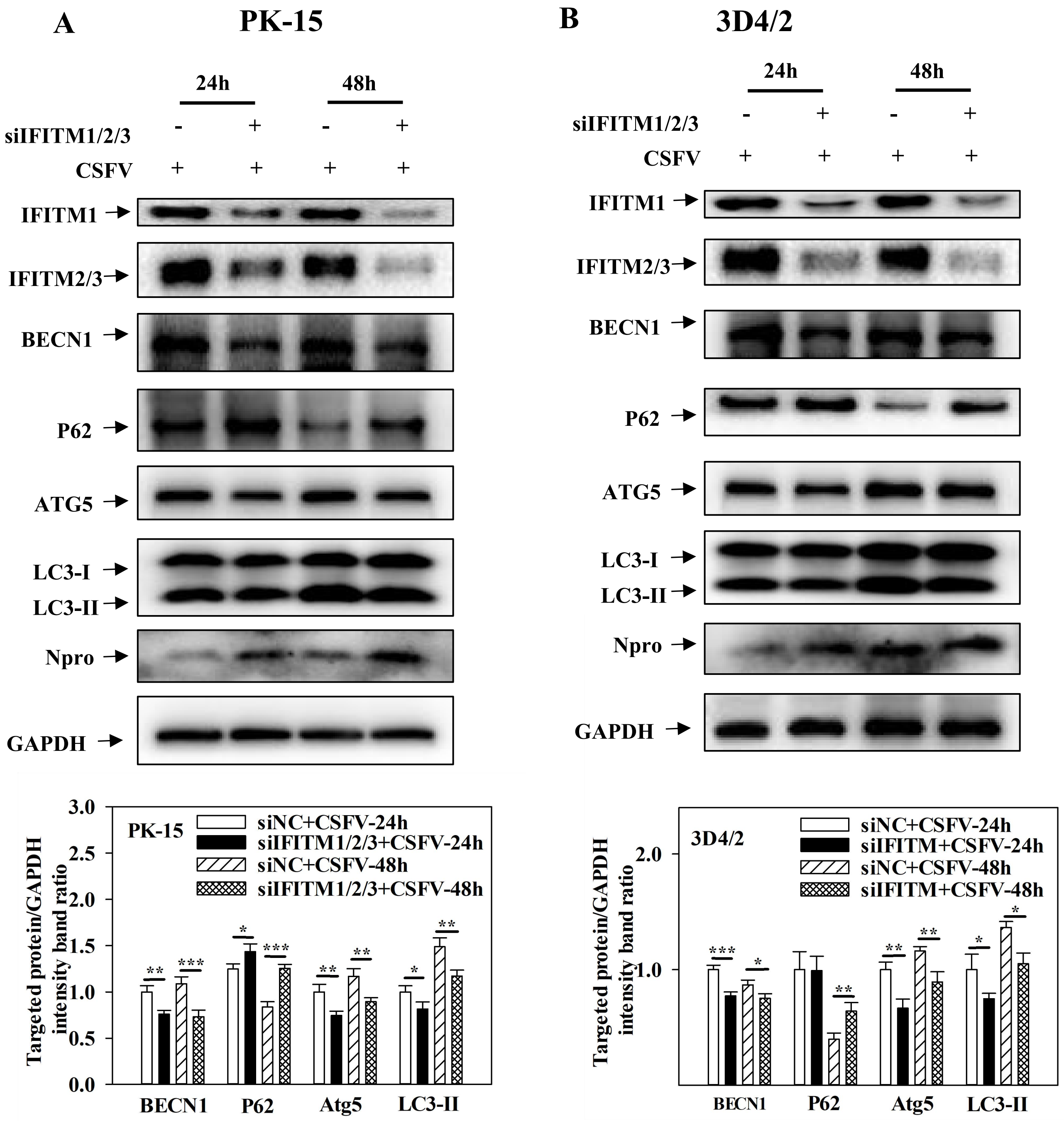

Fig. S6 Silencing of IFITM1/2/3 regulates the expression level of autophagic proteins in CSFV infected PK-15 and 3D4/2 cells. **(A and B)** The preotein levels of BECN1, P62, LC3-I/II, ATG5, Npro and GAPDH were assayed. PK-15 **(A)** and 3D4/2 **(B)** cells were transfected with HA-IFITM1/2/3 or siIFITM1/2/3, followed by incubated with CSFV (MOI = 0.1) for 24 and 48 h. The level of proteins was carried out using Image-Pro Plus 6.0 software.

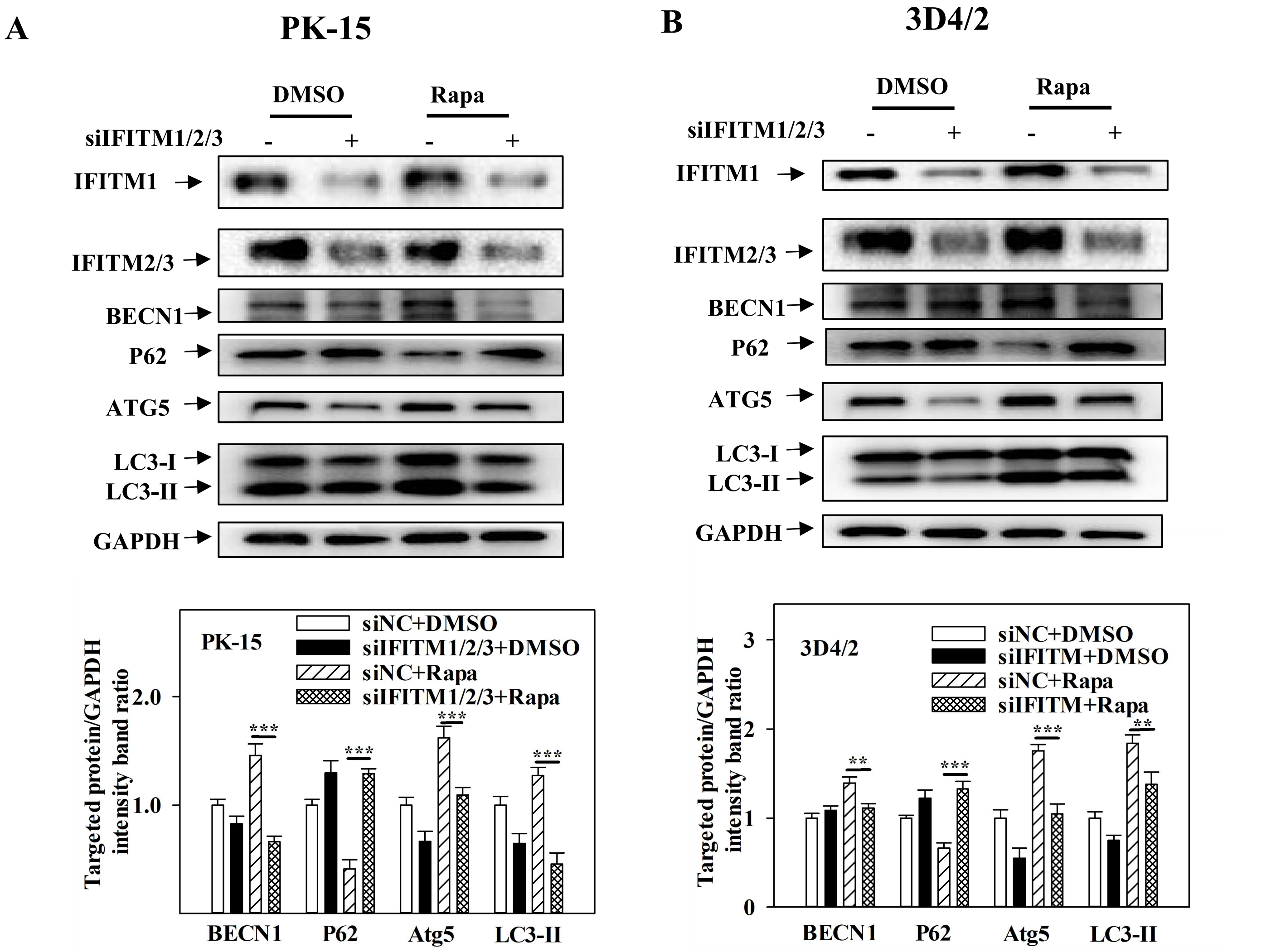

Fig. S7 Silencing of IFITM1/2/3 changes the expression level of autophagic proteins in Rapa treated PK-15 and 3D4/2 cells. **(A and B)** The protein levels of BECN1, P62, LC3-I/II, ATG5 and GAPDH were assayed. PK-15 **(A)** and 3D4/2 **(B)** cells were respectively pretreated with 100 nmol Rapa or equal amount of DMSO for 1 h, followed by transfected with HA-IFITM1/2/3 or siRNA of siIFITM1/2/3 for 24 h. The level of proteins was carried out using Image-Pro Plus 6.0 software.

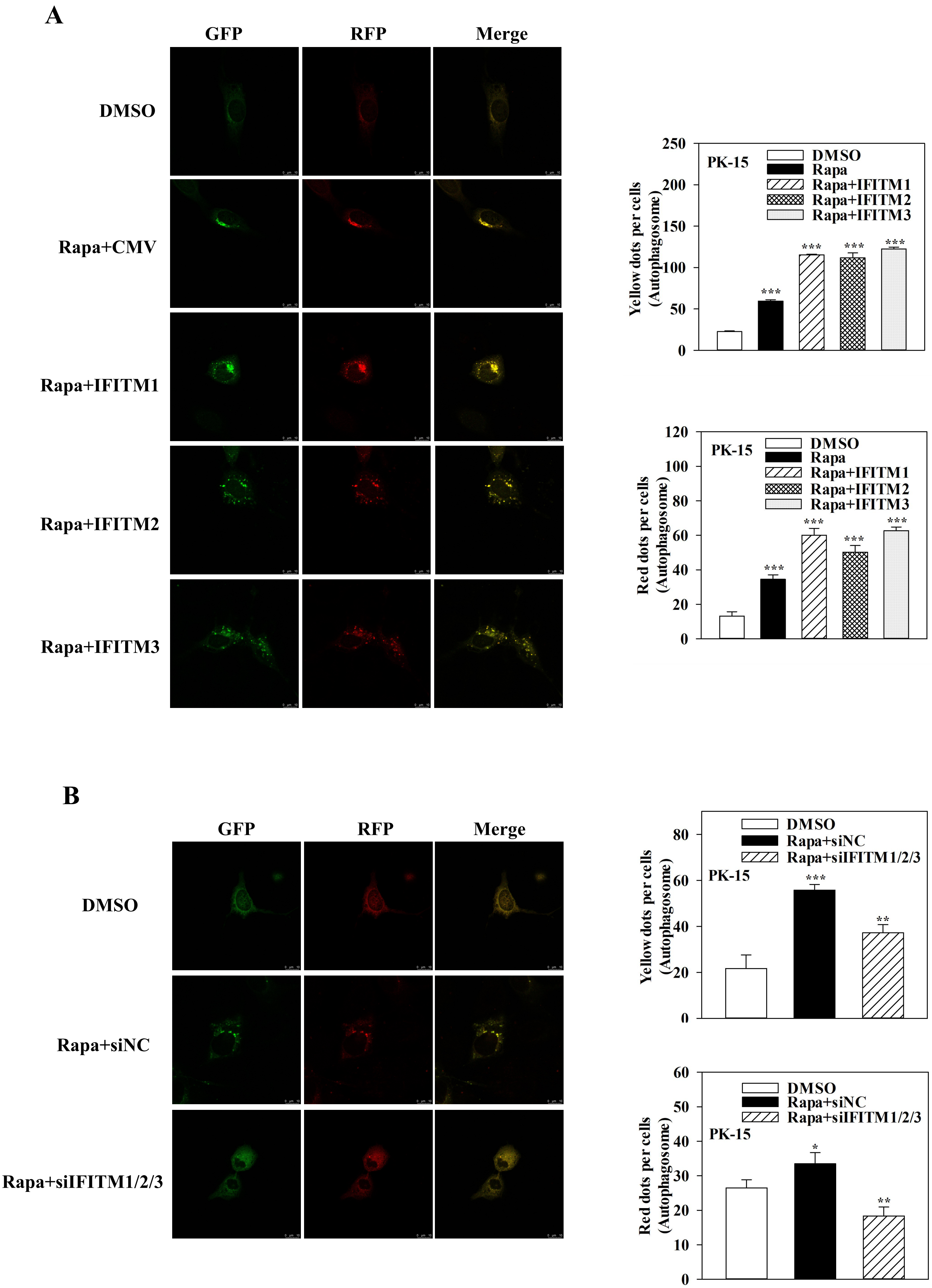
A
GFP
RFP
Merge
DMSO
Rapa+CMV
Rapa+IFITM1
Rapa+IFITM2
Rapa+IFITM3
PK-15
DMSO
Rapa
Rapa+IFITM1
Rapa+IFITM2
Rapa+IFITM3
Yellow dots per cells (Autophagosome)
250
200
150
100
50
0
***
PK-15
Red dots per cells (Autophagosome)
120
100
80
60
40
20
0
***
B
GFP
RFP
Merge
DMSO
Rapa+siNC
Rapa+siIFITM1/2/3
DMSO
Rapa+siNC
Rapa+siIFITM1/2/3
PK-15
Yellow dots per cells (Autophagosome)
80
60
40
20
0
***
**
Red dots per cells (Autophagosome)
60
50
40
30
20
10
0
*
**

Fig. S8 IFITM1/2/3 alter the level of autophagic flux in Rapa treated PK-15 cells. **(A and B)** PK-15 cells were respectively pretreated with 100 nmol Rapa or equal amount of DMSO for 1 h, and then co-transfected mRFP-GFP-LC3 plasmid with HA-IFITM1/2/3 **(A)** or siIFITM1/2/3 **(B)** for 24 h. Confocal fluorescence microscopy was used to capture the yellow dots (autophagosomes) and red dots (autophagolysosomes). The GFP/RFP florescence intensity ratio were analysed using Image-Pro Plus 6.0 software. Scale bar: 10 μm.

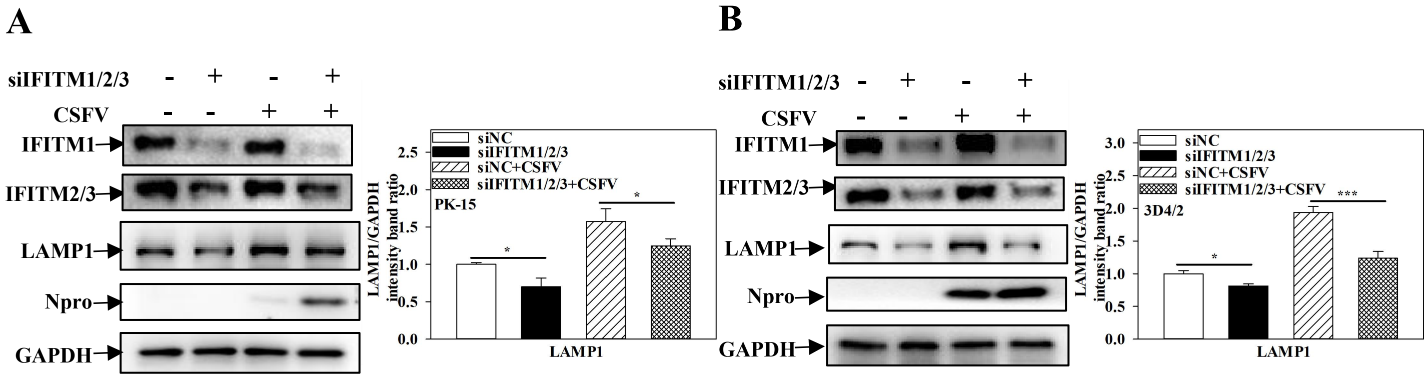

Fig. S9 Silencing of IFITM1/2/3 represses the expression of LAMP1 protein. **(A and B)** PK-15 **(A)** and 3D4/2 **(B)** cells were transfected with siIFITM1/2/3, and then were infected or uninfected with CSFV (MOI = 0.1) for 24 h. The protein levels of LAMP1, Npro and GAPDH were assayed. The level of proteins was carried out using Image-Pro Plus 6.0 software.

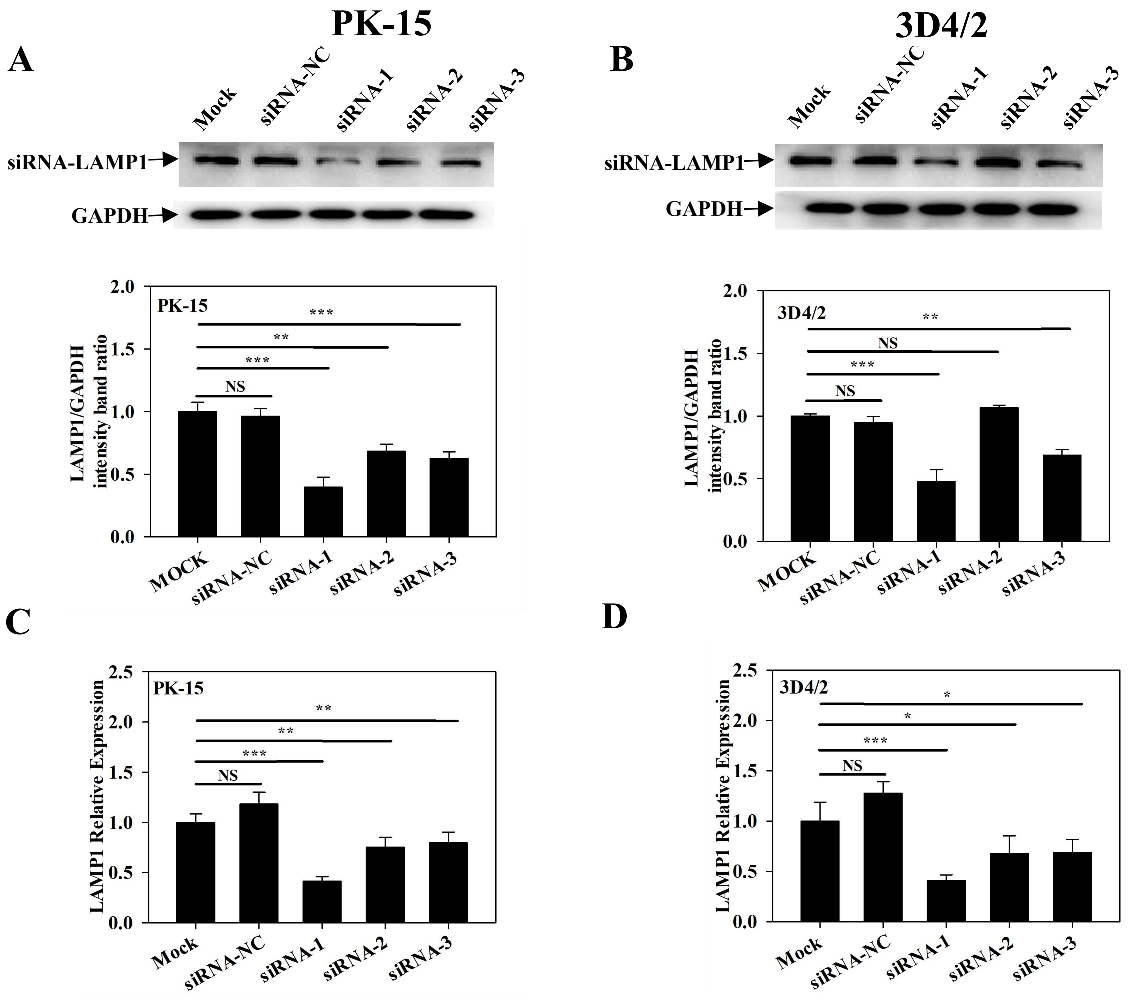

Fig. S10 The siRNA silencing efficiency of LAMP1 in PK-15 and 3D4/2 cells. PK-15 **(A, C)** and 3D4/2 **(B, D)** cells were transfected with siNC or LAMP1 siRNA for 24 h. The siRNA silencing efficiency of LAMP1 were assessed by Western blot analysis **(A, B)** and qRT-PCR **(C, D)**.

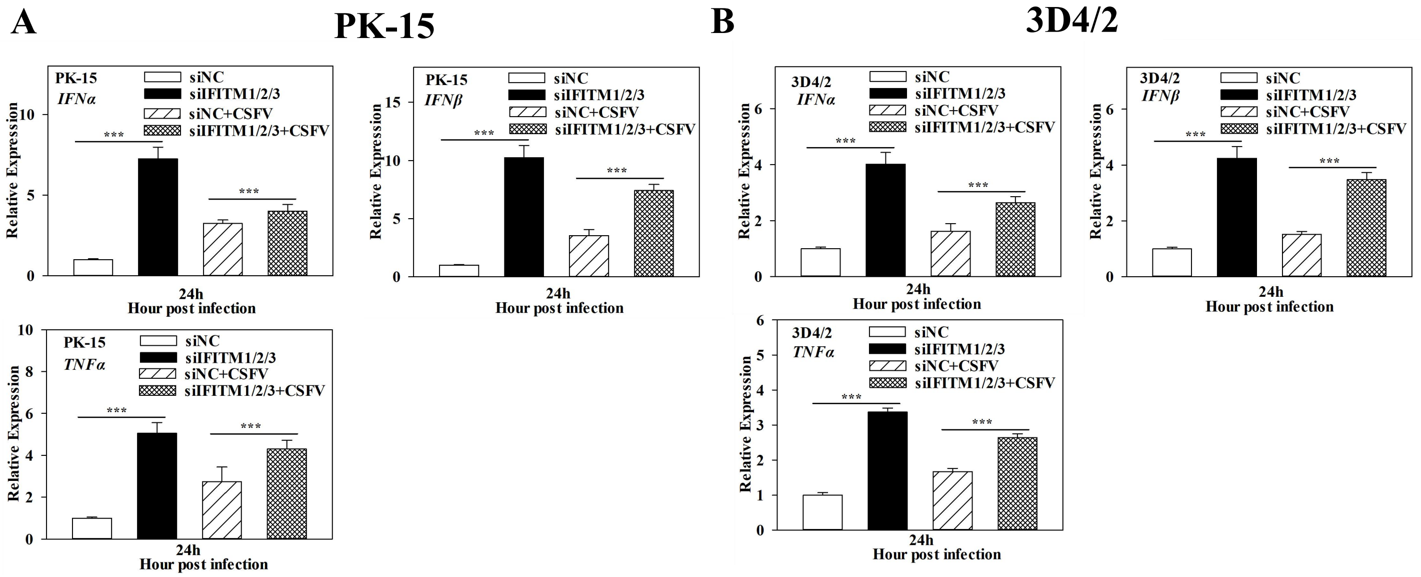

Fig. S11 The transcription level of *IFNα, IFNβ and TNFα* were tesed by RT-qPCR. PK-15 **(A)** and 3D4/2 **(B)** cells were transfected with siIFITM1/2/3, and then were infected or uninfected with CSFV (MOI = 0.1) for 24 h.

Table S1 Primers and siRNA used in this study.

Primer	Forward primer (5’-3’)	Experiment
Flag-SERINC5-F	CTTGCGGCCGCGAATTCAATGTCAGCGCAGTGCTGTGC	Gene clone
Flag-SERINC5-R	TCTAGAGTCGACTGGTACCGATCACACGGAGAACTGGGGAG	
HA-IFITM1-F	GTTCCAGATTACGCTGAATTCATGATCAAGAGCCAGCACGA	Gene clone
HA-IFITM1-R	TCGAGGCATGCCCGGGTACCCTAGCCTCTGTTACTCTTTGCG	
HA-IFITM2-F	GTTCCAGATTACGCTGAATTCATGAACTGCGCTTCCCAGC	Gene clone
HA-IFITM2-R	TCGAGGCATGCCCGGGTACCCTAGCCTCTGTTACTXTTTGCGC	
HA-IFITM3-F	GTTCCAGATTACGCTGAATTCATGAATTGCGCTTCCCAGC	Gene clone
HA-IFITM3-R	TCGAGGCATGCCCGGGTACCCTAGCCTCTGTAATCCTTTATGAGCT	
GAPDH-F	TGGAGTCCACTGGTGTCTTCAC	RT-qPCR
GAPDH-R	TTCACGCCCATCACAAACA	
IFITM1-F	CAAGAGCCAGCACGAGAT	RT-qPCR
IFITM1-R	CAGTTCAGGAAGAGGGTGTT	
IFITM2/3-F	GTCGTCTGGTCCCTGTTCAAC	RT-qPCR
IFITM2/3-R	GAGTAGGCGAAAGCCACGAA	
IFNα-F	CATCCTGGCTGTGAGGAAATA	RT-qPCR
IFNα-R	CAGGTTTCTGGAGGAAGAGAAG	
IFNβ-F	AGCAGATCTTCGGCATTCTC	RT-qPCR
IFNβ-R	GTCATCCATCTGCCCATCAA	
TNFα-F	CACGTTGTAGCCAATGTCAAAG	RT-qPCR
TNFα-R	GAAGAGGACCTGGGAGTAGAT	
LAMP1-F	TTTGGAAGAGGACACACACTC	RT-qPCR
LAMP1-R	GAACTCTGCGTCTGACAGATTA	
pGBKT7-SERINC5-F	ATGGCCATGGAGGCCGAATTCATGATGTCAGCGCAGTGCTGTGC	Y2H
pGBKT7-SERINC5-R	TGCGGCCGCTGCAGGTCGACGTCACACGGAGAACTGGGGAG	
pGBKT7-IFITM1-F	ATGGCCATGGAGGCCGAATTCATGATCAAGAGCCAGCACGA	Y2H
pGBKT7-IFITM1-R	TGCGGCCGCTGCAGGTCGACGCTAGCCTCTGTTACTCTTTGCG	
pGBKT7-IFITM2-F	ATGGCCATGGAGGCCGAATTCATGAACTGCGCTTCCCAGC	Y2H
pGBKT7-IFITM2-R	TGCGGCCGCTGCAGGTCGACGCTAGCCTCTGTTACTCTTTGCGC	
pGBKT7-IFITM3-F	ATGGCCATGGAGGCCGAATTCATGAATTGCGCTTCCCAGC	Y2H
pGBKT7-IFITM3-R	TGCGGCCGCTGCAGGTCGACGCTAGCCTCTGTAATCCTTTATGAGCT	
pGADT7-SERINC5-F	ATGGCCATGGAGGCCAGTGAATTCATGTCAGCGCAGTGCTGTGC	Y2H
pGADT7-SERINC5-R	TGCAGCTCGAGCTCGATGGATCCCTCACACGGAGAACTGGGGAG	
pGADT7-IFITM1-F	ATGGCCATGGAGGCCAGTGAATTCATGATCAAGAGCCAGCACGA	Y2H
pGADT7-IFITM1-R	TGCAGCTCGAGCTCGATGGATCCCCTAGCCTCTGTTACTCTTTGCG	
pGADT7-IFITM2-F	ATGGCCATGGAGGCCAGTGAATTCATGAACTGCGCTTCCCAGC	Y2H
pGADT7-IFITM2-R	TGCAGCTCGAGCTCGATGGATCCCCTAGCCTCTGTTACTCTTTGCGC	
pGADT7-IFITM3-F	ATGGCCATGGAGGCCAGTGAATTCATGAATTGCGCTTCCCAGC	Y2H
pGADT7-IFITM3-R	TGCAGCTCGAGCTCGATGGATCCCCTAGCCTCTGTAATCCTTTATGAGCT	
siRNA IFITM1/2/3-F	CCCUGUUCAACACCCUCUUTT	siRNA
siRNA IFITM1/2/3-R	AAGAGGGUGUUGAACAGGGTT	
siRNA LAMP1-F	GGUGUUAAACAGCAGCUCUTT	siRNA
siRNA LAMP1-R	AGAGCUGCUGUUUAACACCTT	
siRNA NC-F	UUCUCCGAACGUGUCACGUTT	siRNA
siRNA NC-R	ACGUGACACGUUCGGAGAATT	
